# Alkaloids from *Waltheria* spp. (Malvaceae): Chemosystematic Aspects, Biosynthesis, Total Synthesis, and Biological Activities

**DOI:** 10.3390/ijms252413659

**Published:** 2024-12-20

**Authors:** Raquel de M. Silva, Guilherme S. Caleffi, Fernando Cotinguiba

**Affiliations:** Instituto de Pesquisas de Produtos Naturais Walter Mors (IPPN), Centro de Ciências da Saúde (CCS), Universidade Federal do Rio de Janeiro (UFRJ), Avenida Carlos Chagas Filho, 373, Bloco H, Rio de Janeiro 21941-599, RJ, Brazil; raquel_demedeiros@hotmail.com (R.d.M.S.); guilherme.caleffi@ippn.ufrj.br (G.S.C.)

**Keywords:** *Waltheria*, alkaloids, waltheriones, antidesmone, natural products

## Abstract

*Waltheria*, a genus within the Malvaceae family, is abundantly distributed in tropical and subtropical areas worldwide. Many species of this genus are widely utilized in various ways, including chewing, in folk medicine, acting as an anti-inflammatory agent, and treating gastrointestinal disorders, rheumatism, and asthma, among other conditions. These applications are largely due to their secondary metabolites, primarily quinolone alkaloids and cyclopeptides. Several biological activities have been reported for *Waltheria* species, including antifungal, anticancer, trypanocidal, acetylcholinesterase inhibitory, potential anti-HIV, antinociceptive, analgesic, anti-inflammatory, antibacterial, antioxidant, and leishmanicidal activities. This review not only presents information on isolated alkaloids and their biological activities but also delves into biosynthetic, chemosystematic, medicinal chemistry, and total synthesis aspects. Additionally, the manuscript highlights other applications of alkaloids of the genus, such as a study on their herbicidal activity, which shows significant potential for agricultural use.

## 1. Introduction

*Waltheria* L. is a genus of the Malvaceae family; it comprises 80 species, including both accepted names and synonyms (See List S1, Supplementary Materials) [1,2,3]. These plants are characterized as herbs, shrubs, subshrubs, or small trees, predominantly found in pantropical regions [1,2,4].

*Waltheria* species are known for accumulating 4-quinolone alkaloids and cyclopeptides as nitrogenous compounds. Some species hold significant importance in traditional medicine, with their roots and leaves being extensively used in teas prepared by decoction and infusion, as well as being chewable.

The isolation of these alkaloids and investigation of their biological activities have garnered attention from synthetic and medicinal chemists aiming to explore the structural potential of these substances. Some synthetic approaches have been successful in obtaining synthetic and semi-synthetic alkaloids. Many questions about the biosynthesis pathways of 4-quinolone alkaloids still require considerable attention from the scientific community and need to be answered.

This review aims to provide a comprehensive overview of the chemical structures of nitrogenous compounds isolated from the genus *Waltheria*, along with a review of their biological activities, total synthesis, and biosynthesis pathways.

## 2. Traditional Medicine

Several species of *Waltheria* are widely used in traditional medicine across various countries. Notable among them are *Waltheria indica* L., *Waltheria brachypetala* Turks, *Waltheria communis* A. St. Hil, *Waltheria douradinha*, St. Hilaire, and *Waltheria viscosissima* A. St. Hil [5].

*Waltheria indica*, commonly known as velvet leaf, monkey bush, marshmallow, boater bush, buff coat, and leather coat, has numerous local names reflecting its global distribution. The species name “*indica*” associates it with India, where it is called ‘Nallabenda’ in Telugu and ‘Shengali-poondu’ in Tamil [6]. This species is extensively used in traditional medicine for a variety of illnesses. For skin diseases, it is applied as a decoction, used to wash wounds, or applied locally in powdered form. In the treatment of general disorders affecting the whole body, it is administered in the form of an aqueous extract, powder, decoction as tea, maceration, juice, instilling, maceration (with *Piliostigma thonningii*) for local applications.

For nervous system disorders, it is primarily used as a decoction; for digestive system disorders, it is treated with decoctions, crushed, gargling, chewing, decoction with roots of *Securidaca longipedunculata*, infusion, gargling, topical baths (oral route), and chewed by mothers for infants; for respiratory system disorders, it is managed using decoctions, macerations, chewing, juice, leaves, and buds pounded and mixed with water, and decoctions combined with other plants; for blood disorders, it is used as an aqueous extract, chewed, infused, or in decoction form; for reproductive disorders, it is treated with aqueous extracts, juice, infusions, and decoctions; and for urinary disorders, it is addressed primarily with decoctions [7].

*W. indica* is widely used by ethnic groups in Nigeria for treating a variety of ailments, including wounds, ulcers, colds, cough, sore throat, asthma, fever, inflammation, gingivitis, conjunctivitis, malaria, rheumatism, cancer, dysentery, hemorrhoids, leprosy, epilepsy, syphilis, bladder diseases, erectile dysfunction and impotence, anemia, and as a blood tonic. Additionally, traditional healers in Nigeria, Burkina Faso, Mexico, and Panama use *W. indica* to treat diarrhea [7,8,9,10]. In Nigeria and Niger, traditional healers also administer the whole plant to cattle as a tonic against “Nagana”, or animal trypanosomiasis, a disease caused by protozoa of the genus *Trypanosoma brucei* [9,10,11].

In Hawaii, *W. indica* is considered one of the ten most recognized medicinal plant species, and every part of the plant is considered a traditional “aspirin-like” remedy against inflammation and asthma, and the bark of its root is chewed to treat sore throats [7,10,12]. The root is also chewed by the people of Bissa to soothe infections of the gums and teeth; by the people of Mossi in Burkina Faso, it is used to combat coughs, and in India and Jamaica, it is used to treat internal bleeding [7].

According to the South African National Biodiversity Institute (SANBI), *W. indica* leaves are used as a leafy vegetable by the Vavhenda people of South Africa, and the plant is employed to enhance fertility among the Shangaan women. In Limpopo, South Africa, the roots, leaves, and whole plants were used to combat sexually transmitted infections, urinary tract infections, and various childhood illnesses [13].

In Brazil, *W. indica* leaves are also used as a tea to treat inflammation, such as gingivitis [12]. Ethnopharmacological data on *W. viscosissima* indicate that this plant possesses several bioactive properties, including pain control, anti-inflammatory, antifungal, and hypoglycemic properties. In Brazilian popular medicine, its antitussive and expectorant activities are particularly notable [5].

Despite the large number of *Waltheria* species, few have been studied from a chemical perspective. Its extensive use in folk medicine suggests the presence of various chemical groups with pharmacological activities, such as flavonoids, alkaloids, terpenes, sterols, tannins, cardiac glycosides, saponins, anthraquinones, and carbohydrates. Among these, cyclopeptide alkaloids and quinolone alkaloids are the main classes of secondary metabolites already reported in this genus [9].

## 3. Alkaloids from *Waltheria*

Alkaloids are naturally occurring nitrogenous organic compounds known for their significant biological activities. Within the genus *Waltheria*, a diverse group of plants in the Malvaceae family, several types of alkaloids have been identified, particularly on 4-quinolone and cyclopeptide alkaloids. These compounds have garnered interest due to their potential pharmacological properties.

### 3.1. Quinolone Alkaloids

**Figure 1:**
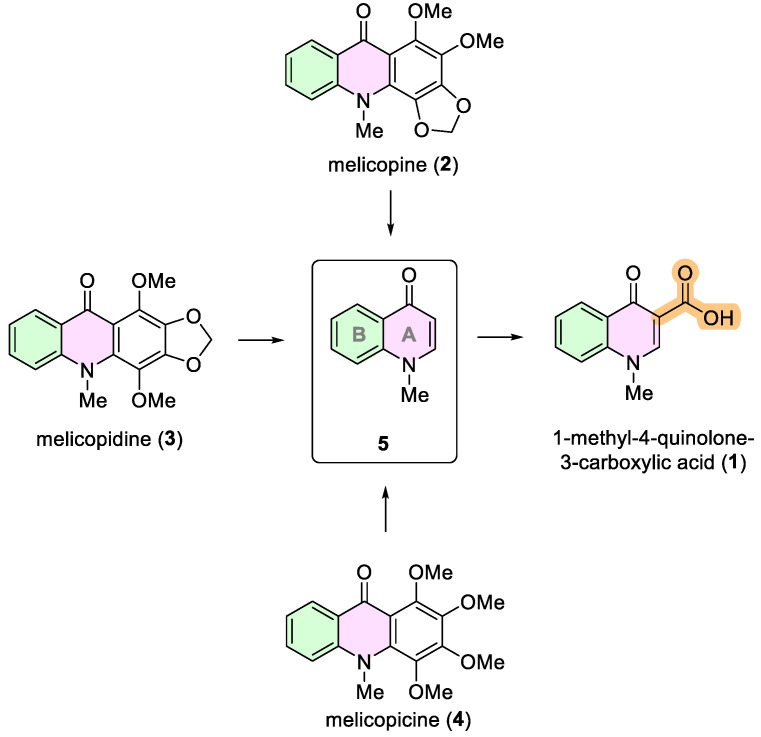
Degradation reaction of the alkaloids melicopine (**2**), melicopidine (**3**), and melicopicine (**4**), resulting in the formation of 1-methyl-4-quinolone (**5**). The corresponding acid, 1-methyl-4-quinolone-3-carboxylic acid (**1**), represents the first compound in the quinolone group (Adapted from Crow and Price, 1949 [14]).

The term “quinolone” was first introduced in 1949 by Crow and Price to describe an alkaloid derivative. This compound, 1-methyl-4-quinolone-3-carboxylic acid (**1**), lacks biological activity and is formed through the degradation reactions of the alkaloids melicopine (**2**), melicopidine (**3**), and melicopicine (**4**), resulting in 1-methyl-4-quinolone (**5**), which subsequently forms the carboxylic acid derivative (Figure 1) [14].

**Figure 2:**
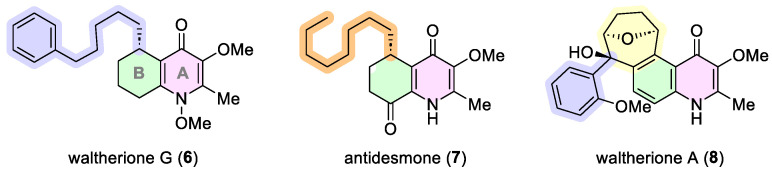
Examples of 4-quinolone alkaloids: phenyl-terminal open chain (waltherione G (**6**)), methyl-terminal open chain (antidesmone (**7**)), and cyclized (waltherione A (**8**)).

The quinolone alkaloids, including the waltheriones and their analogs, are structurally characterized by a common core: the ortho 4-oxo-1,4-dihydropyridine-3-carboxylic acid, which is condensed with another ring that may or may not be aromatic. This core structure is known as 4-oxo-1,4-quinoline or simply 4-quinolone. It features a nitrogen atom as the heteroatom in an aromatic heterocycle and a ketone group at the 4-position. These alkaloids typically have a methoxy group at the C-3 position, and most also possess a methyl group at the C-2 position [14,15]. Recently, Liu et al. reported new alkaloids of this class, termed walindicaones [16]. The 4-quinolone alkaloids can be categorized into phenyl-terminal open chain, methyl-terminal open chain, or cyclized forms (Figure 2).

**Figure 3:**
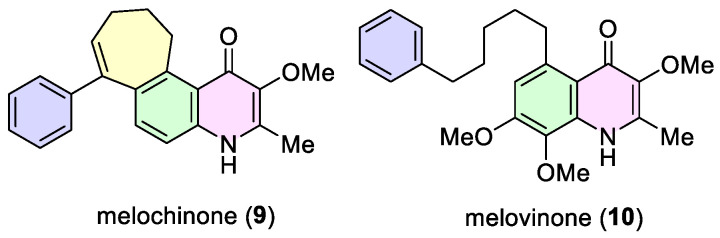
4-quinolone alkaloids melochinone (**9**) and melovinone (**10**).

The first isolation of a 4-quinolone alkaloid from the Malvaceae family was achieved by Kapadia and collaborators in 1975. They isolated melochinone (**9**) from the species *Melochia tomentosa* L. Notably, this compound has not been isolated from *Waltheria* genus until the present review. In 1978, Kapadia et al. isolated melovinone (**10**), which is considered an open-chain analog of melochinone (**9**) (Figure 3) [17,18].

Among the open-chain alkaloids are antidesmone (**7**) and its analogs, which serve as chemosystematic markers of the Antidesmeae tribe within the Phyllanthaceae (Euphorbiaceae) family [19,20,21]. Chemosystematic markers are specific chemical compounds or classes of compounds that serve as tools for understanding the evolutionary relationships and taxonomic classification of plants and other organisms. These markers are often secondary metabolites, such as alkaloids, that are biosynthetically related and may reflect genetic or evolutionary traits. However, antidesmone (**7**) has already been reported in *Melochia chamaedrys* [22], *W. douradinha* [23], and *W. indica* [6,9,10,16,24,25]. Chamaedrone (**11**), which had its first isolation in *M. chamaedrys* [22], has also been reported in *W. indica* [6] and *W. brachypetala* [26].

Among the cyclized alkaloids, the waltherione A (**8**) stands out due to its structural difference from melochinone (**9**), featuring an ether bridge that forms a bicycle [3.2.1] ring system. Waltherione A was first reported in the genus *Waltheria* in *W. douradinha* [23,27], and later, in *W. brachypetala* [28], *W. indica* [6,10,11,29], and *W. viscosissima* [30]. Waltherione B (**12**) has been identified in *W. douradinha* [23], *W. indica* [6], and *W. viscosissima* [30], while waltherione C (**13**) has been reported only in *W. indica*, despite its first isolation from *Melochia odorata* [6,10,11,16,25,29,31].

All structures of the quinolone alkaloids isolated from *Waltheria* species can be found in Table S1 (Supplementary Materials).

### 3.2. Cyclopeptide Alkaloids

This class of alkaloids was first named by Païs et al. in 1963 as peptide alkaloids [32]. In 1975, Tschesche proposed the term “cyclopeptide alkaloid” as a more fitting designation due to the presence of a macrocycle ring in their structure. Cyclopeptide alkaloids are defined by this macrocycle ring and are classified based on the number of atoms in the ring, typically comprising thirteen, fourteen, or fifteen members. The fourteen-member group is the most prominent, as it includes the largest number of isolated alkaloids [33,34,35].

**Figure 4:**
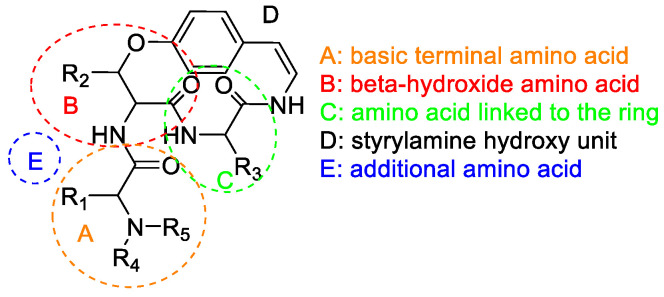
Basic structure of cyclopeptide alkaloids (adapted from Gehm et al., 2022 [36]).

Cyclopeptide alkaloids are a group of polyamide bases composed of four structural units: A, B, C, and D. Unit A is an *N*-mono or dimethylated basic terminal amino acid; unit B is a β-hydroxy amino acid (3-hydroxyproline, 3-hydroxyleucine, or 3-hydroxyphenylserine); the C unit is a ring-bound amino acid; the D unit is a *p*- or *m*-hydroxy styryl amine derived from tyrosine, forming an α-amino acid fragment. Occasionally, an additional unit, E, may be present between units A and B, consisting of an intermediate amino acid (Figure 4) [33,34,35,36,37].

**Figure 5:**
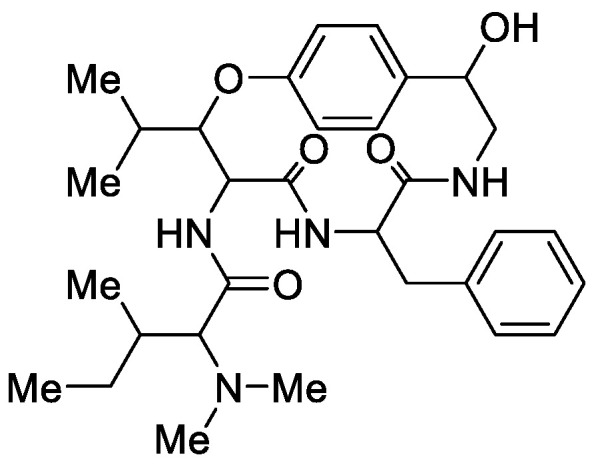
Pandamine (**17**) isolated from *Panda oleosa* (Pandaceae).

Païs and collaborators were the first to discover cyclopeptide alkaloids in 1963, isolating adouetines -X (**14**), -Y (**15**), and -Z (**16**) from *Waltheria americana* (syn. *W. indica*). Their structures were reported in 1968. The first fully elucidated cyclopeptide alkaloid is considered to be pandamine (**17**), which was isolated from *Panda oleosa* (Pandaceae) and had its structure completely determined in 1966 (Figure 5) [32,33,34,38].

All structures of the cyclopeptide alkaloids isolated from *Waltheria* species can be found in Table S2 (Supplementary Materials).

## 4. Chemosystematic Aspects

Cyclopeptide alkaloids and 4-quinolone alkaloids are found in both the genus *Waltheria* and *Melochia*, which belong to the Malvaceae family (Hermannieae tribe). The 4-quinolone alkaloid antidesmone (**7**) has been reported in *M. chamaedrys* [39], *W. douradinha* [36], and *W. indica* [8,11,13,29,37,38]. In 2002, Buske et al. also reported antidesmone in species of *Antidesma* spp. that belong to the Phyllanthaceae (Euphorbiaceae) family (Antidesmeae tribe) as well as in *Hyeronima alchorneoides* and *Thecacoris stenopetala* (all subtribe Antidesminae) [21]. Melochinone (**9**) and melovinone (**10**), natural analogs of antidesmone (**7**), were reported in 1975 and 1978, respectively, by Kapadia and collaborators in the roots of *M. tomentosa* [17,18]. Chamaedrone (**11**) and other analogs of antidesmone (**7**) have been reported in the roots of *M. chamaedrys* [22].

Waltherione A (**8**) has been reported in *W. douradinha* [23,27], *W. indica* [6,10,11,29], *W. viscosissima* [30], *W. brachypetala* [28], *M. chamaedrys* [39], and *M. odorata* [31,40]. Waltherione B (**12**) has also been reported in *W. douradinha* [23], *W. indica* [6], *W. viscosissima* [30], and *M. odorata* [31], while waltherione C (**13**) has been reported in *W. indica* [6,10,11,16,25,29], *M. odorata* [31], and *M. umbellata* [41].

In a previous study by our research group, the chemical profile of *W. indica* was examined through dereplication analysis using UPLC-MS/MS, with data acquisition facilitated by chemoinformatics tools. The preprocessed data were submitted to the GNPS to create a feature-based molecular network (FBMN). This analysis annotated thirty-three 4-quinolone alkaloids in the extracts and fractions of stems and roots and twelve in the extracts and fractions of flowers and leaves. Among these alkaloids, twenty-five were annotated for the first time in a species collected in Brazilian territory. This represents the first chemical investigation of this species and genus utilizing UPLC-Q-TOF-MS/MS analysis in combination with a molecular network approach [42], thus reinforcing the presence of these genus markers. The desreplication of secondary metabolites, particularly alkaloids, plays a crucial role in natural products’ chemistry. This process enables the rapid identification of known compounds within complex samples, which is essential for optimizing resources and focusing research efforts on the discovery and characterization of novel compounds. By minimizing redundant analysis of already identified metabolites, desreplication streamlines the study of natural products, facilitating the exploration of their chemical diversity and potential applications in pharmaceutical, agricultural, and other biotechnological fields.

The cyclopeptide alkaloid adouetine X (**14**), reported in *W. americana* [19,20], has also been found in *M. chamaedrys* [39]. All this information indicates a metabolic relationship between the genera *Waltheria* and *Melochia* and species of the order Antidesmeae of the Phyllanthaceae (Euphorbiaceae) family.

## 5. Biosynthesis

### 5.1. Biosynthesis of 4-quinolone Alkaloids

**Scheme 1:**
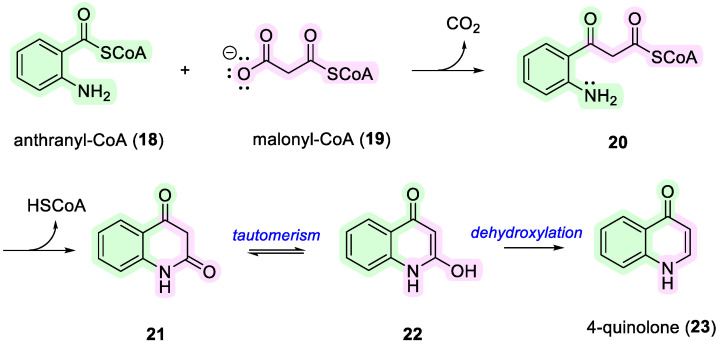
Formation of the 4-quinolone nucleus (**23**) via anthranyl-CoA (**18**) and malonyl-CoA (**19**) (adapted from Dewick, 2009 [43]).

Dewick proposes that the quinolone nucleus forms from anthranilic acid through a series of reactions. Initially, a Claisen condensation initially occurs between anthranyl-CoA (**18**) and malonyl-CoA (**19**). This reaction is driven by the decarboxylation of the malonyl unit, which then undergoes nucleophilic attack by the CH_2_ anion of the malonyl-CoA unit on the thioester carbonyl of anthranyl-CoA. Subsequently, the nitrogen electron pair attacks the carbonyl, releasing an HS-CoA unit. This is followed by tautomerism and dehydroxylation (Scheme 1) [43].

**Scheme 2:**
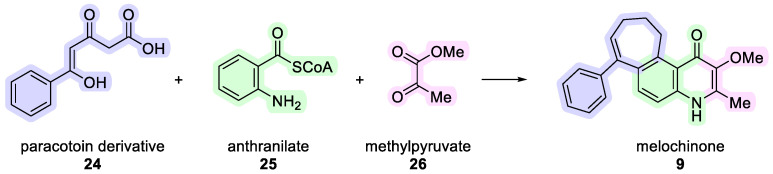
Melochinone biosynthesis proposal (adapted from Kapadia et al., 1975 [17]).

Kapadia et al. noted that the 4-quinolone alkaloid melochinone (**9**) has an unusual structure, featuring a seven-membered ring fused to an aromatic ring. This was the first report of a quinolin-4-one nucleus with a methyl group at position 2 and a methoxyl group at position 3. The authors considered the presence of the seven-membered ring as a biosynthetic puzzle, a problem in biosynthetic terms, suggesting a possible condensation of paracotoin (**24**) with anthranilate (**25**). The presence of methoxyl at position 3 and methyl at position 2 was suggested from the condensation of the anthranilate unit with methyl pyruvate (**26**) (Scheme 2) [17]. However, this proposal lost its meaning after the publication of the biosynthesis of antidesmone (**7**) by Bringmann and collaborators [19].

**Scheme 3:**
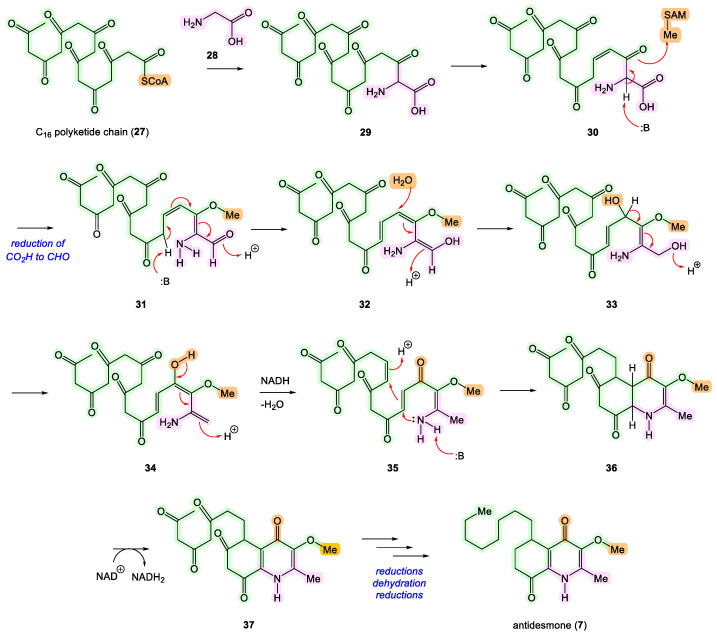
Antidesmone (**7**) biosynthesis (adapted from Bringmann et al., 2000 [19]).

Bringmann and collaborators found the biosynthesis of antidesmone (**7**) to be unusual, prompting them to conduct a study via isotopic labeling with cultures of *Antidesma membranaceum* cells (Phyllanthaceae/Euphorbiaceae). Through the incorporation of precursors labeled with ^13^C (glucose and acetate) and ^15^N (glycine and aspartate), and nuclear magnetic resonance (NMR) analysis, they concluded that antidesmone (**7**) was biosynthesized from a C_16_ polyketide chain (**27**) and glycine (**28**). They also suggested an alternative biosynthetic route. The methyl at carbon 2 of the 4-quinolone system originates from the amino acid glycine (**28**), while for the methoxy at carbon 3 derives its oxygen from the acetate pathway and the methyl from S-adenosylmethionine (SAM). The authors proposed that antidesmone (**7**) and melochinone (**9**) might share a common precursor (Scheme 3) [19].

Alkaloids can be biosynthesized in different ways across plant families due to variations in biosynthetic pathways, specific enzymes, and genetic regulations present in each group [44]. Waltheriones, for instance, exhibit an uncommon biosynthetic pathway, with only a limited number of experimental studies reported. Notably, previously proposed biosynthetic pathways for this class of alkaloids remain unverified through experimental approaches, highlighting a significant gap in understanding. Furthermore, no comprehensive biosynthetic investigations of waltheriones within the genus *Waltheria* had been conducted prior to this review.

### 5.2. Biosynthesis of Cyclopeptide Alkaloids

Based on current knowledge, all peptides synthesized by plants are ribosomal. They are produced from messenger RNA, undergo post-translational modifications, and are proteolytically cleaved to yield post-translationally modified peptides (RiPPs) [45].

**Figure 6:**
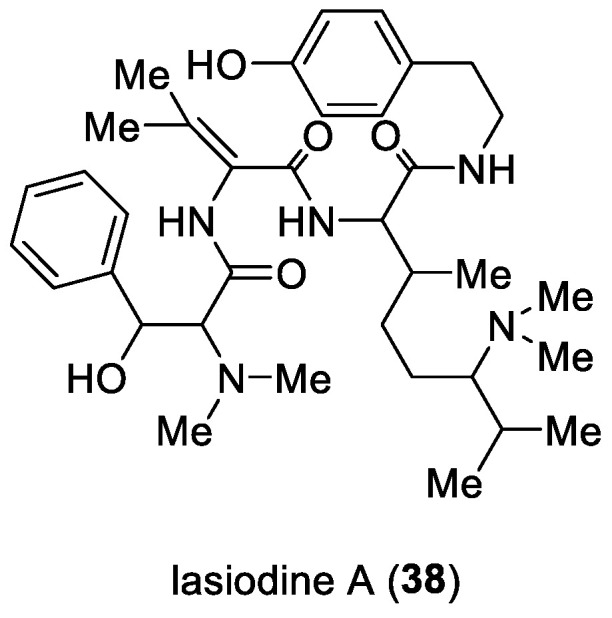
Lasiodine A (**38**) (adapted from Marchand et al., 1969 [46]).

Despite this understanding, knowledge about the biosynthetic pathway of cyclopeptide alkaloids in plants is limited. However, investigations using likely intermediaries have allowed researchers to propose some main routes. One such route was elucidated through the isolation of linear cyclopeptide alkaloids, such as lasiodine A (**38**), which features a free phenolic group and a dihydroamino acid unit (Figure 6) [46].

**Scheme 4:**
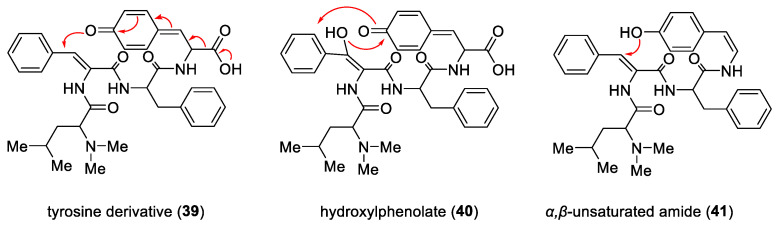
Probable precursors of cyclopeptide alkaloids. Curved arrows (*red*) indicate probable formation of the macrocycle (adapted from Warnhoff, 1971; Bhat et al., 1987 [47,48]).

Quinone may serve as a possible precursor, consisting of a dihydrophenylalanine-phenylalanine linked to an oxidized tyrosine (**39**). In the biosynthetic pathway of 13- or 14-membered alkaloids, a decarboxylation followed by a Michael addition can lead to the formation of the macrocycle. Another possibility involves the attack of a β-hydroxyl group on the quinone carbonyl, followed by decarboxylation and the subsequent formation of hydroxylphenolate (**40**). Alternatively, the phenolic hydroxyl group may attack the β-carbon of the α, β-unsaturated amide (**41**) (Scheme 4) [47,48].

**Figure 7:**
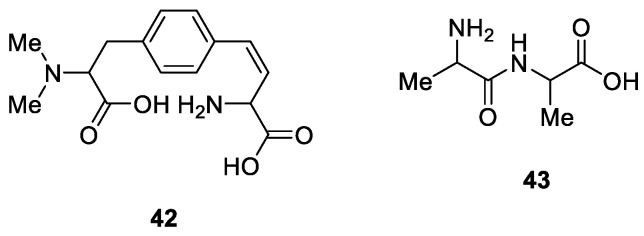
*p*-phenylenedialanine (**42**) and its dihydrogenated counterpart (**43**) (adapted from Schmidt et al., 1985 [49]).

The linkage between the benzene ring and the 15-membered cyclopeptide alkaloids, which lack the β-phenoxyamine acid component, is established through carbon atoms. A possible biosynthetic pathway for these alkaloids might involve the *p*-phenylenedialanine unit (**42**) or its dihydrogenated form (**43**) as precursors (Figure 7) [49].

Recent advances using transcriptome mining approaches have revealed connections between cyclopeptide alkaloids in *Ceanothus americanus* and RiPP (ribosomally synthesized and post-translationally modified peptide) precursor peptides, along with the identification of gene clusters containing split BURP peptide cyclases. These findings were further substantiated by the isolation of novel cyclopeptides, such as arabipeptins from *Coffea arabica*, and the enzymatic reconstitution of BURP activity, providing critical insights into their biosynthesis. However, despite decades of research, their biosynthetic mechanisms remain largely enigmatic. Significant gaps persist in the understanding of the biosynthetic pathways underlying cyclopeptide alkaloids, highlighting the need for more comprehensive studies in this field [50].

## 6. Total Synthesis

The intriguing molecular structures of alkaloids isolated from *Waltheria* stand in stark contrast to the limited research on their total syntheses reported in the literature. To the best of our knowledge, there have been no reported total syntheses of the cyclopeptide alkaloids from *Waltheria* species (Table S2). However, attention is growing toward the synthesis of alkaloids with a quinolin-4(1*H*)-one skeleton (Table S1).

**Scheme 5:**
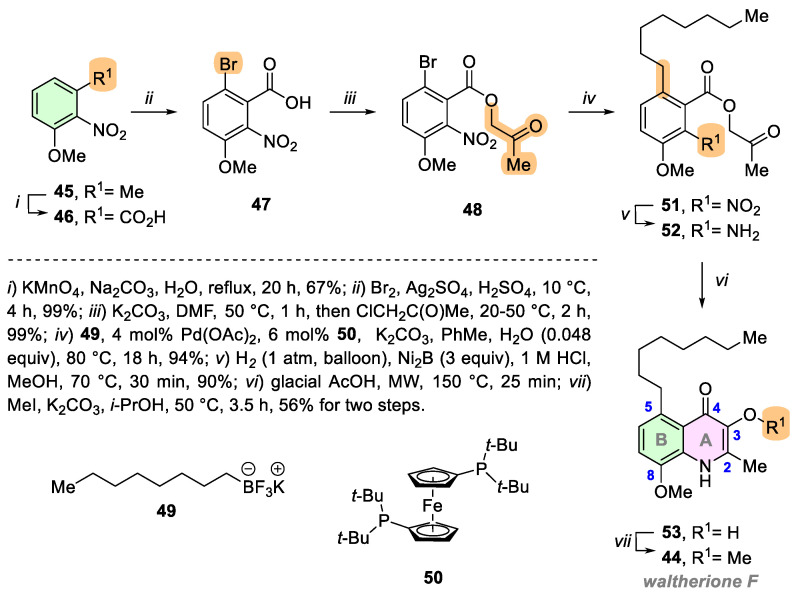
Kaufman and Larghi’s total synthesis of waltherione F (**44**).

In 2018, Kaufman and Larghi’s group reported the first total synthesis of waltherione F (**44**) (Scheme 5) [51]. Like most quinolin-4(1*H*)-one products from *Waltheria*, this natural compound features a 2-methyl-3-methoxy substitution at the A-ring. Notably, it bears an *n*-octyl side chain and a methoxy group at the B-ring’s 5 and 8 positions, respectively.

The authors begin the synthetic route from commercially available anisol **45**. First, it undergoes oxidation with KMnO_4_, followed by bromination using Br₂ in H_2_SO_4_ catalyzed by Ag_2_SO_4_, yielding compound **47** with a good yield (66% over two steps). Next, the reaction between compound **47** and chloroacetone under basic conditions produces the key intermediate **48** with an excellent yield (99%). Subsequently, the *n*-octyl group was introduced via an efficient palladium-catalyzed Suzuki–Miyaura C*sp*^2^-C*sp*^3^ cross-coupling reaction [52,53,54,55] between the bromoarene **48** and the alkyltrifluoroborate salt **49**. The sterically hindered 1,1′-bis(di-*t*-butylphosphino) ferrocene (**50**); ligand was employed to afford **51** in 94% yield. Then, nickel boride promoted the chemoselective reduction in the nitro group under an H_2_ atmosphere, leading to the formation of anthranilate **52** with a 90% yield (Scheme 5).

The heterocyclic A-ring was obtained from intermediate **52** through a Niementowski-type cyclization (formation of cyclic carbon chains)/rearrangement step at 150 °C under microwave irradiation using glacial acetic acid, resulting in **65**. Finally, a methylation step of the 3-hydroxy group of **65** afforded waltherione F (**44**) in 56% yield over the two steps (Scheme 5).

**Scheme 6:**
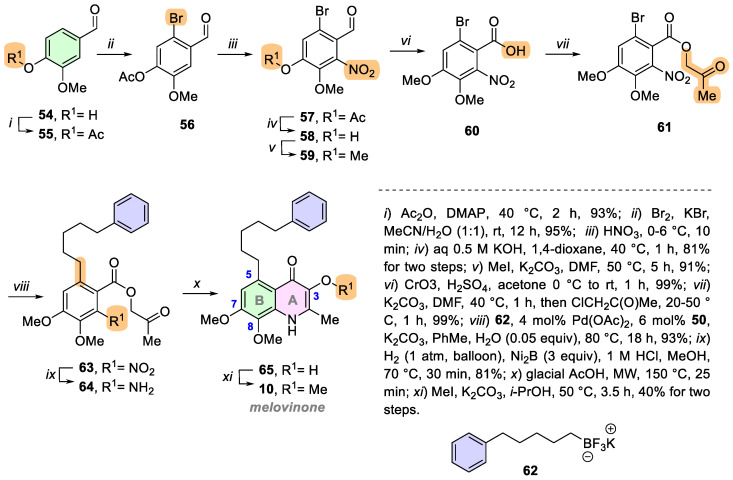
Kaufman and Larghi’s total synthesis of melovinone (**10**).

In the following year, Kaufman and Larghi’s group expanded their strategy to the total synthesis of melovinone (**10**) (Scheme 6) [56]. This natural alkaloid is distinguished by an extra methoxy group at the B-ring’s 7-position and a 5-phenylpentyl group at the 5-position, compared to compound **44**. For this synthesis, vanillin (**54**) was chosen as the starting material. Initially, **54** was acetylated, then selectively subjected to bromination and nitration. Subsequent steps included hydrolysis of the acetate and methylation of the phenolic group, yielding compound **59**. The aldehyde group in **59** was then oxidized to benzoic acid (**60**) using Jones’ reagent. Finally, compound **60** was reacted with chloroacetone under basic conditions to produce the key intermediate **61** with a 99% yield (Scheme 6).

Subsequently, the successful attachment of the 5-phenylpentyl group was accomplished using the established palladium-catalyzed Suzuki–Miyaura [51]. The bromoarene **61** reacted with potassium trifluoro(5-phenylpentyl)borate **62** to yield compound **63** with a high efficiency of 93%, demonstrating the effectiveness of this cross-coupling method for introducing alkyl side chains (Scheme 6). Notably, the alkyltrifluoroborate salt **62** was synthesized from the commercial (2-bromoethyl)benzene through a four-step process (details not depicted). The final stages in synthesizing melovinone (**10**) mirrored the previously established protocols for waltherione F (**44**), involving a nickel-catalyzed selective nitro reduction, Niementowski type cyclization/rearrangement, and methylation, culminating in a 32% yield of melovinone (**10**) over these steps (Scheme 6).

Finally, the synthetic strategy developed by Kaufman and Larghi’s group allowed for obtaining the natural quinolin-4(1*H*)-ones waltherione F (**44**) and melovinone (**10**). These compounds were synthesized through synthetic routes consisting of seven steps with an overall yield of 31%, and 11 steps with an overall yield of 18%, respectively [51,56].

**Scheme 7:**
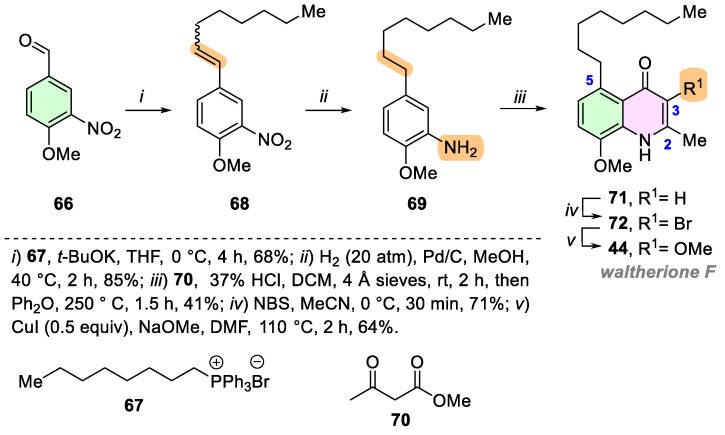
Cox’s total synthesis of waltherione F (**44**).

In 2019, Cox’s group reported the total synthesis of waltherione F (**44**) and its analogs using the Conrad–Limpach synthesis of the quinolin-4(1*H*)-one scaffold as the key step (Scheme 7) [57]. The commercially available 4-methoxy-3-nitrobenzaldehyde (**66**) underwent a Witting reaction [58] with octyltriphenylphosphonium bromide (**67**), yielding olefin **68** at a 68% yield. Subsequently, a one-pot reduction in both the nitro group and the C=C bond of **68** using Pd/C under 20 atm of H_2_ produced aniline **69** with an 85% yield (Scheme 7).

The reaction between the aniline **69** and the ketone group of the b-ketoester **70** under acid conditions at room temperature generated an imine-enol tautomer intermediate. Upon heating at 250 °C using diphenyl ether as a solvent, the substrate cyclized to form the 2-methylquinolin-4(1*H*)-one derivative **71** in 41% yield after 1.5 h. Finally, the introduction of the 3-methoxy group occurred through two steps: bromination followed by methoxylation catalyzed by copper(I) iodide (Scheme 7). As a result, waltherione F (**44**) was obtained via a five-step route with an 11% overall yield, and its structure was confirmed by X-ray diffraction data. Additionally, this strategy facilitated the synthesis of other derivatives by modifying substituents at the 2- and/or 3-position. By using different β-ketoesters or sodium ethoxide in the last step, new compounds suitable for screening in medicinal chemistry programs were obtained.

**Scheme 8:**
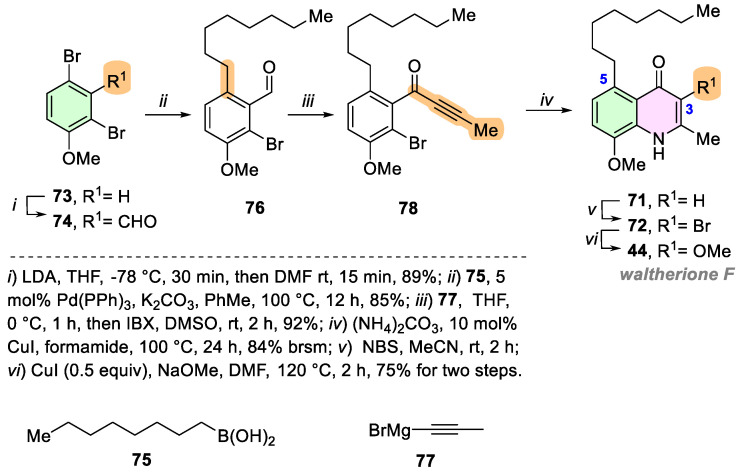
Pabbaraja and Mehta’s total synthesis of waltherione F (**44**).

In 2020, Pabbaraja and Mehta’s group reported the total synthesis of waltherione F (**44**) using an orthogonal ammonia insertion into an *ortho-*bromoaryl ynone intermediate (Scheme 8) [59]. The authors began with the commercially available 2,4-dibromoanisole (**73**), which underwent regioselective formylation [60], followed by the Pd-catalyzed Suzuki–Miyaura [54,55] with the alkyl boronic acid **75**, yielding compound **76** in 85% yield. Next, a Grignard addition of propargylmagnesium bromide **77**, followed by oxidation with 2-iodoxybenzoic acid (IBX), produced the key intermediate 2-bromoaryl ynone **78** in 92% yield (Scheme 8).

The reaction between **78** and ammonium carbonate, using copper(I) iodide as a catalyst in formamide at 100 °C, led to the formation of 2-methylquinolin-4(1*H*)-one **71** in 84% yield based on the recovered starting material (brsm) (Scheme 8). In this one-pot transformation, the ammonium carbonate acted as both a base and a source of ammonia, via a tandem Michael addition followed by Cu(I)-mediated ArCsp^2^-N coupling [59,61]. Subsequently, following conditions adapted from Cox’s previous work [57], compound **71** underwent bromination at the 3-position, followed by methoxylation, resulting in the synthesis of waltherione F (**44**) in 75% over these two steps (Scheme 8).

Finally, waltherione F (**44**) was obtained via a six-step route with an overall yield of 33%. The efficiency and convenience of this method were demonstrated for other 2-methylquinolin-4(1*H*)-ones obtained through orthogonal ammonia insertion into *ortho-*haloaryl ynones.

**Scheme 9:**
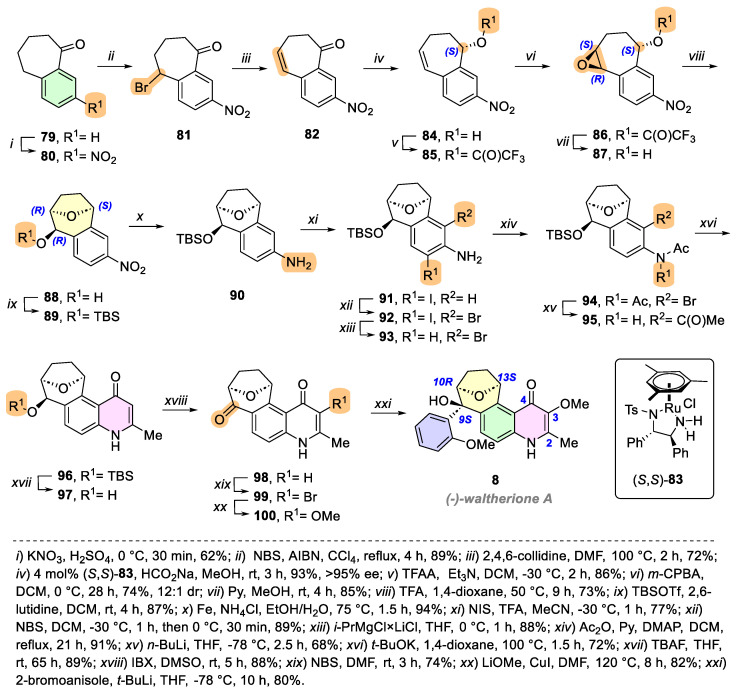
Nakagawa-Goto’s total synthesis of waltherione A (**8**).

Recently, Nakagawa-Goto’s group reported the total synthesis of waltherione A (**8**), a quinolin-4(1*H*)-one alkaloid isolated from *Waltheria* species (Scheme 9) [62]. This natural product shows a complex structure in which the 2-methyl-3-methoxyquinolin-4(1*H*)-one skeleton is fused with an oxabicyclo [3.2.1] octane, bearing three stereogenic centers.

The synthetic route began with tetrahydro-5*H*-benzo [7] annulen-5-one **79**, which underwent nitration using potassium nitrate and sulfuric acid, followed by the benzylic bromination with *N*-bromosuccinimide (NBS) and azobisisobutyronitrile (AIBN) as a radical initiator. Subsequently, the 2,4,6-collidine promoted the formation of the olefin compound **82** after 2 h at 100 °C in DMF (Scheme 9).

To install the first stereogenic center, the authors employed an asymmetric transfer hydrogenation reaction of the carbonyl group, using the Noyori-Ikariya ruthenium (*S*,*S*)-**83** complex [63,64]. By combining sodium formate as the hydrogen source with the Ru(II)-complex under mild and near-neutral conditions [65,66,67], they achieved the alcohol (*S*)-**84** in 93% yield and excellent enantioselectivity (>95% ee). Next, to selectively obtain the *anti*-epoxide **87**, protection of the benzylic alcohol (*S*)-**84** with trifluoroacetate was necessary (Scheme 9).

Subsequently, an intramolecular epoxide ring opening promoted by trifluoracetic acid (TFA) at 50 °C in 1,4-dioxane, led to the formation of the oxabicyclo [3.2.1] octane **88** in 73% yield after 9 h. The benzylic alcohol was protected with the *t*-butyl(dimethyl)silyl (TBS) group, and then the nitroarene was reduced to aniline **90** using Fe-NH_4_Cl as a reducing agent, in 94% yield (Scheme 9) [68].

To obtain the desired intermediate **93** (R^1^ = H and R^2^ = Br), a three-step halogenation sequence was necessary. First, iodation occurred at the least hindered position with *N*-iodosuccinimide (NIS), followed by bromination with NBS. The removal of iodine was achieved with the Grignard reagent *i*-PrMgCl·LiCl, resulting in compound **93** in 60% yield over three steps [69]. Next, a diacetylation of the aniline **93** gave the product **94**, which underwent a Fries-like intramolecular rearrangement [70] using *n*-BuLi at −78 °C, yielding the *o*-acylaminoacetophenone derivative **95** with a 72% yield (Scheme 9).

After optimization of the conditions for the Camps cyclization [71], the 2-methylquinolin-4(1*H*)-one **96** was obtained with 72% yield using *t*-BuOK as a base in 1,4-dioxane at 100 °C. Deprotection of the TBS group, followed by oxidation with IBX in DMSO at room temperature, afforded compound **98** in 74% yield. Then, as reported previously for the synthesis of waltherione F (**44**), the 3-methoxy group was introduced in a two-step sequence of bromination followed by methoxylation, yielding compound **100**. Finally, treatment of 2-bromoanilose with *t*-BuLi in THF at −78 °C generates the organolithium reagent in situ for the stereoselective 1,2-addition to carbonyl group of **100**. This process selectively proceeded through the substrate`s convex face, yielding waltherione A (**8**) in an 80% yield.

Therefore, the total synthesis of waltherione A (**8**) was achieved in 21 steps with a 1.2% overall yield. The spectroscopic and optical rotational data were identical to the isolated compound, confirming the absolute configuration of waltherione A (**8**) as 9*S*, 10*R*, and 13*S*.

In summary, the total synthesis of only three alkaloids isolated from *Waltheria* has been reported to date in the literature: waltherione A (**8**), melovinone (**10**), and waltherione F (**44**). The synthesis of waltherione F (**44**) has been achieved through three different synthetic strategies, involving five to seven steps, and yielding the natural product in 11–33% global yield on a small scale (11 to 68 mg).

The formation of the heterocyclic A-ring represents the most significant challenge in these syntheses. This step exhibited the lowest yield in the strategies based on Niementowski-type cyclization/rearrangement (Scheme 5) or Conrad–Limpach synthesis (Scheme 7). Conversely, a strategy employing a tandem Michael addition of ammonia to *ortho-*haloaryl ynone **78**, followed by Cu(I)-mediated ArCsp^2^-N coupling, enabled efficient heterocyclic A-ring formation with 84% yield for this step (Scheme 8).

These findings underscore the need for developing more efficient and scalable synthetic approaches to access a broader range of Waltheria alkaloids, especially those with more complex structures, such as waltherione A (**8**), which contains stereogenic centers.

## 7. Pharmacological Properties of Alkaloids

Significant biological activities have been reported for this class of 4-quinolones, including antifungal activities [6,24,40], anticancer [10], trypanocide [9,11,72,73], acetylcholinesterase inhibitors [28], potential anti-HIV activity [31], antinociceptive, analgesic, anti-inflammatory and antioxidant activities [5,7,10,12,16,74], leishmanicidal activity, and antibacterial activity [6].

In 2007, Emile and collaborators first reported the biological activity of the alkaloid waltherione A (**8**), demonstrating antifungal activity against *Candida albicans*, *Cryptococcus neoformans*, and *Saccharomyces cerevisiae* at a concentration of 50 µg mL^−1^ [40]. In 2016, Cretton and collaborators assessed the antifungal activity of 21 compounds isolated from *W. indica*, collected in Nigeria against *Candida albicans*. Among these, ten 4-quinolone alkaloids—waltherione N (**101**), (*R*)-vanessine (**102**), waltherione Q (**103**), 8-deoxoantidesmone (**104**), antidesmone (**7**), waltherione E (**105**), waltherione G (**6**), waltherione I (**106**), waltherione J (**107**), and, waltherione F (**44**)—exhibited increased inhibitory activity in both planktonic cells and biofilms (MIC ≤ 32 μg mL^−1^). Notably, waltherione G (**6**) and waltherione J (**107**) showed the broadest spectrum of activity (MIC ≤ 32 μg mL^−1^) against all tested yeast strains, including pathogenic species *Candida glabrata*, *C. krusei*, *C. tropicalis*, *C. parapsilosis*, and the non-pathogenic yeast, *Saccharomyces cerevisiae*) [24].

In 2022, our research group conducted a chemosensitization assay and found that, in the absence of fluconazole, only waltherione G (**6**) inhibited the growth of a mutant strain of *Saccharomyces cerevisiae* overexpressing *Candida albicans* proteins CaMDR1, indicating antifungal activity. In the presence of fluconazole, the growth inhibition of *Candida albicans* was enhanced by waltherione G (**6**), along with waltherione C (**13**), waltherione H (**108**), waltherione J (**107**), waltherione L (**109**), chamaedrone (**11**), and antidesmone (**7**), suggesting a synergistic or additive interaction between these 4-quinolone alkaloids and fluconazole [6].

In 2017, Monteillier, Cretton, and collaborators investigated the cancer chemopreventive activity of Waltheria indica. Both the decoction and alkaloid extract showed significant activity at 20 µg mL^−1^, inhibiting the transcription factor NF-κB by 51% and 79%, respectively. NF-κB is known to promote tumor development and progression and is a key player in inflammation-driven cancers. The isolated compounds from the dichloromethane extract were also tested for their quinone reductase (QR) inducing properties, a complementary strategy in cancer chemoprevention that targets tumor initiation. The alkaloids waltherione A (**8**) and waltherione C (**13**) were identified as major components in the decoction extract and were the two most potent NF-κB inhibitors. The presence of potent NF-κB inhibitors and QR-inducing compounds in the decoction supports the traditional use of W. *indica* in cancer chemoprevention [10].

In 2003, Buske and collaborators patented the metabolite antidesmone (**7**) due to its in vitro activity against the causative agent of Chagas disease, *Trypanosoma cruzi*. Antidesmone demonstrated significant antitrypanosomal (antichagasic) activity, inhibiting trypanosome growth at an initial concentration of 0.37 μg mL^−1^ and an IC_50_ = 0.054 μg mL^−1^, being then an antitrypanosomal (antichagasic) alkaloid, is notably lower than the concentrations required for benznidazole (initial concentration 30 μg mL^−1^; IC_50_ = 0.69 μg mL^−1^) [72]. In 2001, Bringmann et al. published a study showing that antidesmone had an IC_50_ value of 0.02 μg mL^−1^ against *T. cruzi* [74]. In 2014, Cretton et al. reported that the antidesmone (**7**) exhibited the highest and most selective antitrypanosomal activity for *T. cruzi* (IC_50_ = 0.054 μg mL^−1^). Additionally, in a 2015 study, they reported that waltherione C (**13**) had the highest and most selective antitrypanosomal activity for *T. cruzi* (IC_50_ = 0.69 μg mL^−1^) with low cytotoxicity (IC_50_ = 35.17 μg mL^−1^) among the compounds tested [9,11].

In 2009, Lima and collaborators investigated the chemical components of the leaves of *W. brachypetala* Turks and evaluated the inhibition of acetylcholinesterase (AChE) activity by the alkaloids found. Waltherione A (**8**) (IC_50_ = 134.1 ± 20.9 μg mL^−1^), *N*-methyl-waltherione A (**110**) (IC_50_ = 122.7 ± 19.7 μg mL^−1^) in addition to the cyclopeptide alkaloid waltherine A (**111**) (IC_50_ = 113.4 ± 35.2 μg mL^−1^) exhibited significant activities [28].

In 2014, Jadulco and collaborators reported that the 4-quinolone alkaloids isolated from *M. odorata* twigs demonstrated significant activities in an in vitro anti-HIV cytoprotection assay. The concentration of the drug that induces half the maximum effect (EC_50_) was 22.12 μg mL^−1^ for waltherione A (**8**) and 0.29 μg mL^−1^ for waltherione C (**13**). Additionally, these compounds inhibited the formation of HIV capsid protein P2415 formation in infected T cells by more than 50%, with effective concentrations of 0.67 μg mL^−1^ and 0.33 μg mL^−1^, respectively [31].

The initial studies investigating the analgesic and anti-inflammatory activities of *W. indica* extracts showed promising results against inflammation and pain. However, the limited number of doses tested, and the absence of a negative control, prevent definitive conclusions and evidence for the use of traditional pain relievers [7].

In 2016, a study by Yougbare-Ziebrou demonstrated that the aqueous extract of *W. indica* inhibited the DPPH radical, lipoxygenase, and lipid peroxidation, presenting anti-edema, analgesic, and antioxidant effects. These findings support the traditional use of *W. indica* for treating inflammatory diseases [74].

To further examine the affected immune or inflammatory responses modulated by these extracts, Lackzo et al. (2019) used ethanolic extracts of aerial parts of *W. indica* from Nigeria in analyzes of LPS and factor-stimulated human macrophages of tumor necrosis (TNF)-α/IF-γ. The results demonstrated that *Waltheria* extracts inhibited key inflammatory cytokines and cytokine receptors, including levels of interleukin protein IL-1B, IL-1ra, IL-8, and IL-6. Additionally, there was a reduction in mRNA and TNF-α protein levels, as well as the protein levels of its receptor, TNF RII. This suggests a decrease in TNF-α-associated inflammatory signaling, along with a significant reduction in mRNA and nuclear factor kappaB (NF-kB) protein. When NFkB is activated, it promotes the expression of pro-inflammatory genes and increases the levels of the aforementioned cytokines [12].

This principle is also evident in the study by Monteillier et al. (2017), which identified waltherione A (**8**) and C (**13**) as the main components of the dichloromethane extract of aerial parts, both acting as NF-κB inhibitors [10]. In 2022, Viegas et al. clarified that the crude ethanolic extract and the alkaloid fraction of the aerial parts of *W. viscosissima* possess antinociceptive properties in several in vivo experimental studies, indicating potential central and peripheral actions. The bioactive effects are comparable to standard drugs like morphine and dexamethasone, but with the advantage of presenting antioxidant activity and no motor alterations, making it a promising alternative for the treatment of inflammation and pain [75].

In a recent study, Liu and collaborators reported that the alkaloids 8-demethoxywaltherione R (**112**), (5*R*)-waltherione M (**113**), walindicaone F (**114**), walindicaone G (**115**), waltherione G (**6**), walindicaone H (**116**), (11*S*,12*R*,15*S*)-11—dihydroxy-waltherione A (**117**), and waltherione C (**13**) isolated from *W. indica* reduced TNF-α-induced NF-κB activity with IC_50_ values ranging from 7.1 to 12.1 μM, comparable to the positive control (BAY 11-7082, IC_50_ = 9.7 μM). Additionally, the compounds 8-demethoxywaltherione R (**112**), (5*R*)-waltherione M (**113**), (5*S*)-waltherione M (**118**), and waltherione G (**6**) exhibited significant nitric oxide (NO) inhibitory activity with IC_50_ values ranging from 11.0 to 12.8 μM, surpassing the activity of the positive control (L-NMMA, IC_50_ = 22.7 μM) [16].

Our research group also reported excellent leishmanicidal activities for the alkaloids waltherione G (**6**), waltherione H (**108**), waltherione L (**109**), chamaedrone (**11**), and antidesmone (**7**). These alkaloids achieved nearly 100% inhibition of the growth of *Leishmania infantum* at low concentrations [6].

Gressler and collaborators reported that 4-quinolone alkaloid vanessine (**102**) exhibited antibacterial activity against *Escherichia coli* (MIC = 25.0 µg mL^−1^), *Salmonela setubal* (MIC = 50.0 µg mL^−1^), and *Klebsiella pneumoniae* (MIC = 25.0 µg mL^−1^) [23]. Our research group also reported that waltherione P (**119**) (MIC = 3.13 μg mL^−1^) and antidesmone (**7**) (MIC = 6.25 μg mL^−1^) showed the most promising results against *Staphylococcus aureus*. Additionally, only *Staphylococcus epidermidis* was inhibited by the alkaloids waltherione C (**13**), waltherione H (**108**), waltherione J (**107**), waltherione L (**109**), chamaedrone (**11**), waltherione P (**119**), and antidesmone (**7**) [6].

A recent study published by Yabré and collaborators analyzed the hydroethanolic extract of *W. indica* and its fractions, revealing the presence of compounds such as phenolics, flavonoids, tannins, coumarins, sterols/terpenes, and saponins. The study detected a phenolic compound content of 116.03 ± 1.02 mg of tannic acid equivalent (TAE)/g in the residual fraction and 146.62 ± 2.02 mg TAE/g in the hydroethanolic extract, in addition to a significant flavonoid content. The lethal dose was estimated at 5000 mg kg⁻^1^. Furthermore, the extract and its fractions demonstrated strong antioxidant and anti-inflammatory effects, inhibiting enzymes, such as phospholipase A2 and 15-lipoxygenase, scavenging free radicals, reducing ferric ions, inhibiting lipid peroxidation and blocking carrageenan-induced edema [76].

Cyclopeptide alkaloids from the genus *Waltheria* have garnered significant interest due to their antimicrobial potential. Among the compounds tested, adouetin Y (**15**), which can be isolated from *W. americana* [32,38] and *W. douradinha* [77], exhibited notable bactericidal activity with a minimum lethal concentration (MLC = 50 μg mL^−1^) and bacteriostatic activity with a minimum inhibitory concentration (MIC = 25 μg mL^−1^) against *Enterococcus faecium* [78]. These findings underscore the potential of cyclopeptide alkaloids as promising antimicrobial agents, encouraging further research into their therapeutic applications.

## 8. Derivatives of 4-Quinolone Alkaloids

The preparation of synthetic and semi-synthetic derivatives of 4-quinolone alkaloids is actively progressing, reflecting the growing interest of synthetic and medicinal chemists in this class of compounds.

**Figure 8:**
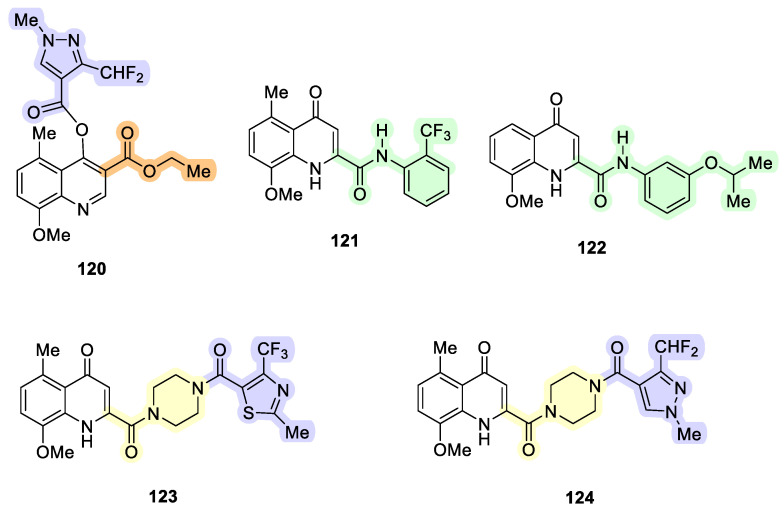
Design strategy of the target compounds: **120** by the introduction of the ester group (*orange*) and pyrazole ring (*lilac*); compounds **121** and **122** by the introduction of an aromatic amide group (*green*); compounds **123** and **124** combining pyrazole ring (*lilac*) and piperazine-amide groups (*yellow*).

Hua et al. (2021) synthesized a series of ester and pyrazole derivatives (Figure 8) based on the waltherione alkaloids’ structure. Among these derivatives, compound **120** demonstrated excellent inhibition against *Physalospora pyricola*, a pathogenic fungus that attacks apples and pears during storage. The assay showed that **120** exhibited activity equivalent to carbendazim and fluopyram at 200 μg mL^−1^. Additionally, microscopy techniques showed that this compound caused damage to the fungus’ vacuoles and cell membranes. Therefore, this derivative holds promise as a potential agent for controlling phytopathogens [79].

In 2022, the same research group reported the preparation of twenty-two derivatives based on the structure of waltherione F (**44**). Similarly to their previous work, the authors monitored antifungal activities against phytopathogens. Compounds **121**, **122,** and **123** were the most active against the rot fungus (*Rhizoctonia solani*), which attacks potato, bean, tobacco, corn, and soybean crops. The strategy for preparing these derivatives involved maintaining the amide groups and introducing piperazines, which were previously identified as enhancing antifungal activity (Figure 8) [80].

**Figure 9:**
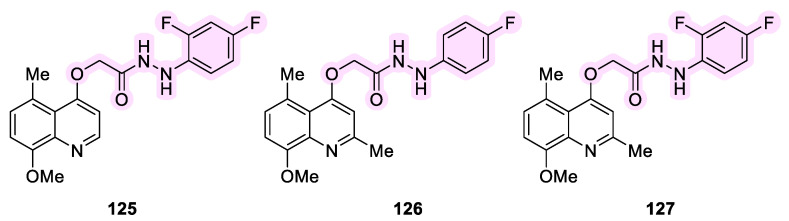
Design strategy of target compounds **125**, **126**, and **127** by the introduction of the acethydrazide group (*pale pink*) and varying the presence of methyl in the quinoline ring.

In 2023, Chen et al. synthesized a series of twenty-three waltherione F (**44**) derivatives containing hydrazides and acethydrazide groups. Tests evaluating the fungicidal potential of these compounds revealed that the acethydrazide derivatives **125**, **126**, and **127** inhibited more than 90% of the growth of the phytopathogen *Fusarium graminearum*, which causes fusarium head blight (FHB). In addition to introducing the hydrazide group, they also varied the quinolone structure by adding or omitting a methyl group at position 4, as shown in Figure 8. Structure-activity relationship (SAR) analysis indicated that the presence of methyl enhanced the fungicidal activity in some cases. Furthermore, the acethydrazide derivatives demonstrated greater potency in in vitro assays than the hydrazide derivatives (Figure 9) [81].

## 9. Other Applications

Although 4-quinolone alkaloids are known for their various biological activities, they also have significant applications in agriculture, particularly as antifungal agents. Liang et al. prepared the dichloromethane extract of *W. indica* stems collected in China, which demonstrated broad-spectrum antifungal activity against eight phytopathogenic fungi from in vitro and in vivo cultures. Notably, it was highly effective against carbendazim-resistant *Sclerotinia sclerotiorum* with an inhibition rate of 100% to 500 μg mL^−1^. Through a bioassay-guided method, two 4-quinolone alkaloids, antidesmone (**7**) and waltherione C (**13**), were isolated and identified as fungicidal compounds. Antidesmone (**7**) exhibited superior fungicidal activities against eight phytopathogenic fungi, outperforming commercial botanical fungicides, such as osthole, carvacrol, and eugenol, with an inhibition rate exceeding 70% at 50 μg mL^−1^ [25].

Antidesmone (**7**) demonstrated significant negative cross-resistance with carbendazim against *Sclerotinia sclerotiorum* for the first time, showing much better antifungal activity (EC_50_ = 0.60 μg mL^−1^) against carbendazim-resistant strains compared to carbendazim-sensitive strains (EC_50_ = 9.61 μg mL^−1^). Furthermore, the dichloromethane extract from *W. indica* stems effectively controlled the development of eleven species of phytopathogenic fungi in vivo. These findings suggest that both the dichloromethane extract of *W. indica* and antidesmone (**7**) have potential as botanical fungicides, particularly for managing carbendazim-resistant fungi [25].

Another notable application is the botanical nematicidal activity of certain alkaloids. Waltherione A (**8**) and waltherione E (**105**), isolated from *Triumfetta grandidens* (Malvaceae), demonstrated significant nematicidal activities against *Meloidogyne incognita*, based on mortality and effect on egg hatching. These compounds showed a high mortality against second-stage juveniles (J2s) of *M. incognita*, with EC_50_ values of 0.09 μg mL^−1^ for waltherione E (**105**) and = 0.27 μg mL^−1^ for waltherione A (**8**) after 48 h. Additionally, they showed a substantial inhibitory effect on egg hatching, with 91.9% inhibition for waltherione E (**105**) and 87.4% for waltherione A (**8**), after 7 days of exposure at a concentration of 1.25 μg mL^−1^ [82]. Later, the same research group published a study where the fractions of ethyl acetate and the alkaloids 5′-methoxy-waltherione A (**128**), waltherione A (**8**), and waltherione C (**13**), isolated from the roots of *W. indica* from Vietnam, were used. The results indicated that 5′-methoxywaltherione A (**128**) and waltherione A (**8**) are highly efficient in controlling plant parasitic nematodes. Furthermore, waltherione C (**13**) exhibited potent nematicidal activity against root-knot nematodes (RKNs) [29].

Sampaio and collaborators investigated the effects of antidesmone (**7**), extracted from *W. brachypetala*, on photosynthesis and plant growth, evaluating its potential as an herbicide. The results showed that antidesmone (**7**) inhibits adenosine triphosphate (ATP) synthesis and non-cyclic electron transport in chloroplasts. Chlorophyll fluorescence revealed that antidesmone (**7**) acts as an inhibitor of the Hill reaction, affecting the donor and receptor sides of photosystem II. In vivo experiments, antidesmone (**7**) reduced dry biomass and significantly inhibited root growth in tomato (*Physalis ixocarpa*) and grass (*Lolium perenne*) seeds, but did not affect germination or stem growth in *L. perenne*, indicating selectivity in herbicide activity. This pioneering study suggests that antidesmone (**7**) could be developed into a new class of herbicides, offering a promising alternative that is safe for agricultural productivity, humans, and the environment [83].

## 10. Conclusions

Among the eighty species of the genus *Waltheria*, few have been the focus of chemical studies and only four have had alkaloid isolation: *W. douradinha*, *W. indica* (syn. *W. americana*), *W. viscosissima,* and *W. brachypetala*. These studies led to the isolation and identification of sixty-one 4-quinolone alkaloids and seven cyclopeptides. These sixty-eight alkaloids have been evaluated against several biological targets, with some demonstrating significant biological activities, including the antifungal, anticancer, trypanocidal, antichagasic, and anti-HIV cytoprotective activities. Despite this progress, our understanding of the biosynthesis of these compounds remains limited. Few experimental studies have been published, and several biosynthetic steps, such as cyclizations and oxidations, remain unclear, contributing to the structural diversity of these alkaloids. The inclusion of Bringmann’s study on 4-quinolones from Phyllanthaceae (Euphorbiaceae) aims to underscore biosynthetic and structural comparisons, providing better contextualization of the relevance of this example. Additionally, there are no molecular-level studies to elucidate the genes and enzymes involved in biosynthesis. On the other hand, research into the total synthesis of alkaloids isolated from *Waltheria* has been garnered, increasing attention within the synthetic chemistry community.

Since the first synthesis of waltherione F (**44**) in 2018, various strategies have been developed to obtain this natural alkaloid, as well as other quinolin-4(1*H*)-ones like melovinone (**10**) and the more structurally complex waltherione A (**8**). However, the synthesis of tetrahydroquinolin-4(1*H*)-ones and cyclopeptide alkaloids isolated from *Waltheria* remains unexplored. Additionally, the preparation of inspired molecules and derivatives of 4-quinolone alkaloids, along with structure-activity relationship (SAR) studies, are still in their infancy. Given the diverse structures of waltheriones and peptides already isolated from *Waltheria* species, there is significant potential for obtaining new semisynthetic derivatives and exploring biological activities. Current research indicates that these derivatives have notable agricultural defensive properties against phytopathogens affecting crops such as wheat, soybeans, corn, apples, and pears. To fully unlock the potential of these complex alkaloid structures for future use in medicinal chemistry screening programs, it is crucial to continuously develop innovative, efficient, and more convergent synthetic approaches.

## Supplementary Materials

The following supporting information can be downloaded at: https://www.mdpi.com/article/10.3390/ijms252413659/s1. References [84,85,86,87,88,89] are cited in the Supplementary Materials.
