# Supplementary material for: Alkaloids from Waltheria spp. (Malvaceae): Chemosystematic Aspects, Biosynthesis, Total Synthesis, and Biological Activities
Source: Int J Mol Sci. 2024 Dec 20;25(24):13659. doi: 10.3390/ijms252413659 (PMC11727749; doi:10.3390/ijms252413659)
Supplement: Supplementary file 1 [file ijms-25-13659-s001.zip › ijms-3347709-supplementary.pdf]

Supplementary Materials

Table S1. 4-quinolone alkaloids from species of *Waltheria*.

Compound	Structure	Name	Species
8	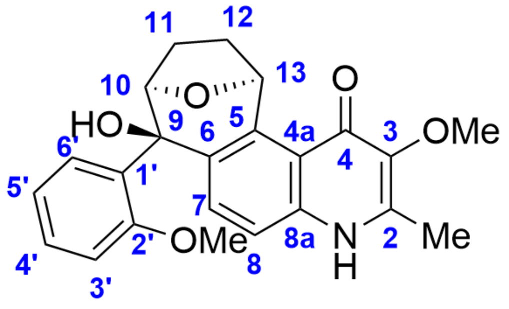	waltherione A	*Waltheria douradinha*[23,27] *Waltheria indica*[6,10,11,29] *Waltheria viscosissima*[30] *Waltheria brachypetala*[28]
128	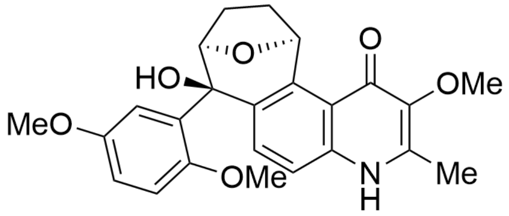	5′-methoxy-waltherione A	*Waltheria indica*[16,29,84]
117	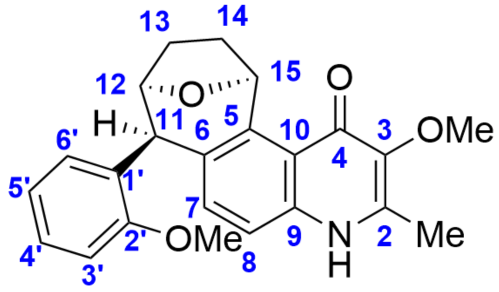	(11*S*,12*R*,15*S*)-11-dehydroxy-waltherione A	*Waltheria indica*[16]
110	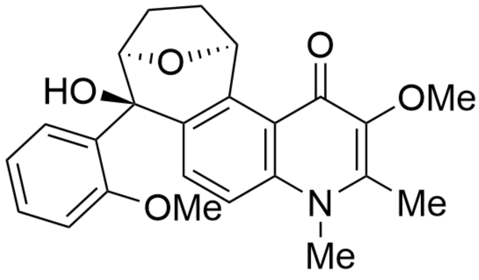	*N*-methyl-waltherione A	*Waltheria brachypetala*[28]
12	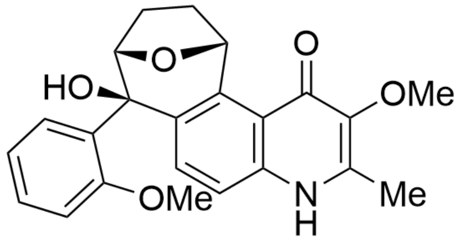	waltherione B	*Waltheria douradinha*[23] *Waltheria indica*[6] *Waltheria viscosissima*[30]

**13**	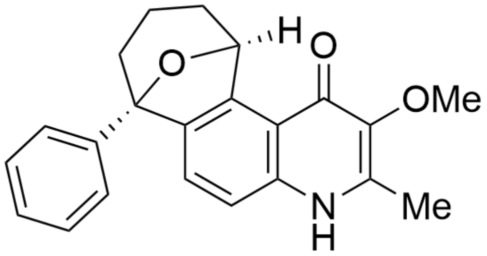	waltherione C	*Waltheria indica*[6,10,11,16,25,29]
**129**	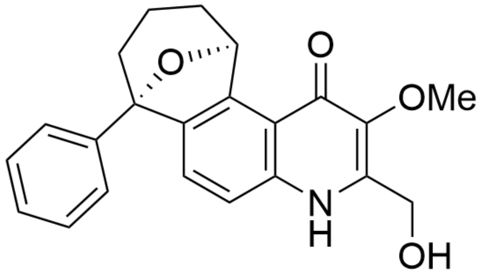	2-hydroxymethyl-waltherione C	*Waltheria indica*[84]
**130**	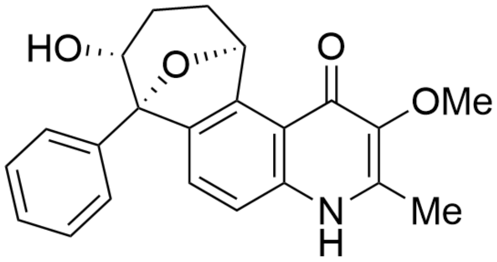	(9*S*,10*S*,13*S*)-10-hydroxy-waltherione C	*Waltheria indica*[84]
**105**	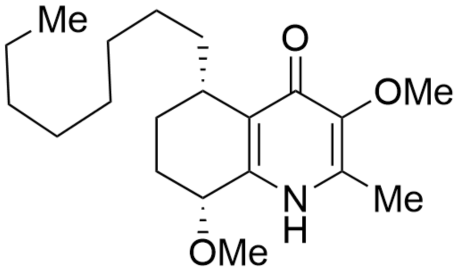	waltherione E	*Waltheria indica*[9,10,16,24]
**44**	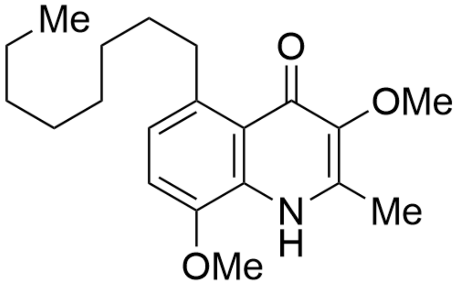	waltherione F	*Waltheria indica*[9,10,24]
**131**	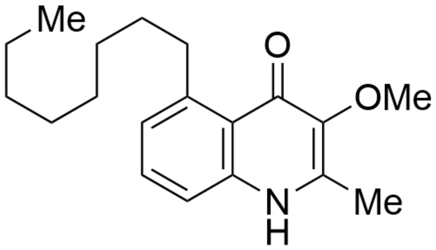	8-demethoxy-waltherione F	*Waltheria indica*[84]

**132**	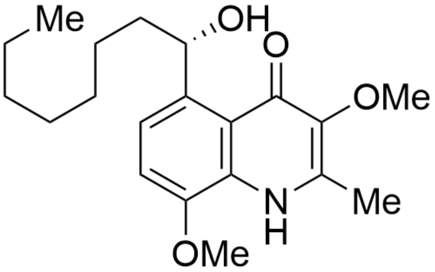	11(*R*)-hydroxy-waltherione F	*Waltheria indica*[16]
**133**	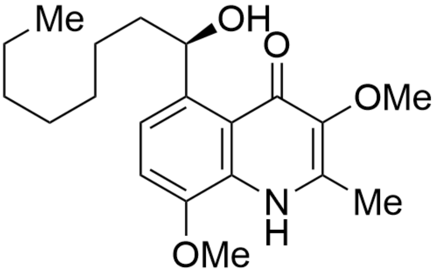	11(S)-hydroxy-waltherione F	*Waltheria indica*[16]
**6**	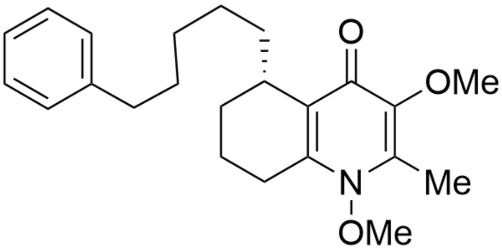	waltherione G	*Waltheria indica*[6,9,10,16,24]
**134**	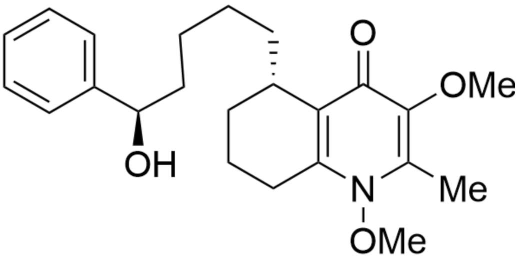	(*S*)15-hydroxywaltherione G	*Waltheria indica*[84]
**108**	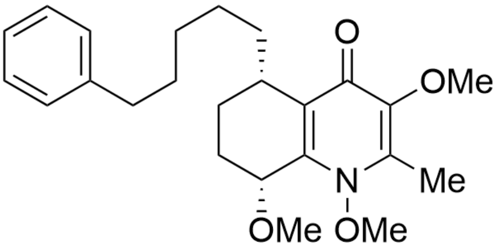	waltherione H	*Waltheria indica*[6,9,10,16,24]
**106**	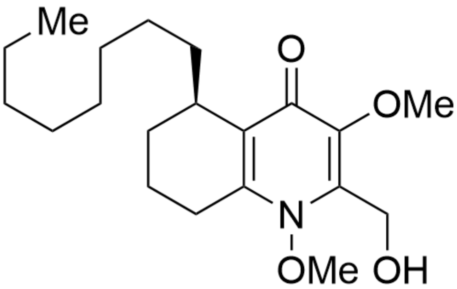	waltherione I	*Waltheria indica*[9,10,24]

**107**	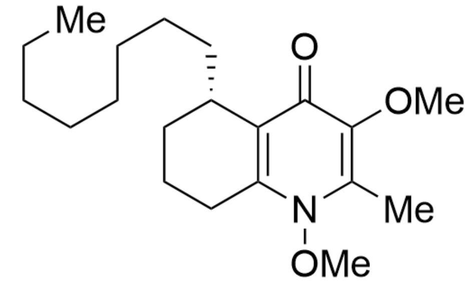	waltherione J	*Waltheria indica*[6,9,10,24]
**135**	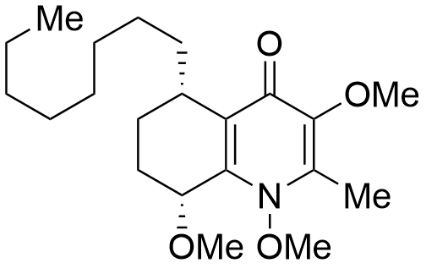	waltherione K	*Waltheria indica*[9,10,24]
**109**	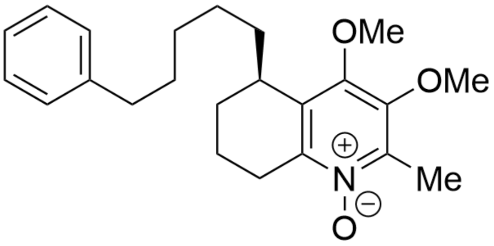	waltherione L	*Waltheria indica*[6,9,10,24]
**118**	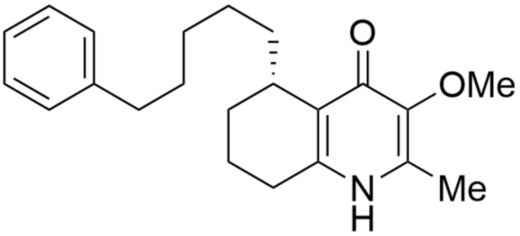	5(*S*)-waltherione M	*Waltheria indica*[10,24]
**113**	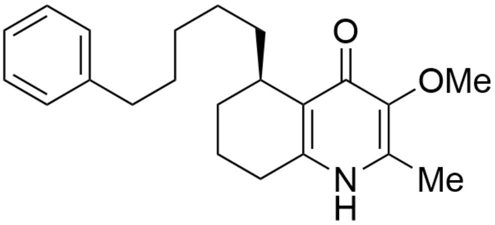	5(*R*)-waltherione M	*Waltheria indica*[16]
**136**	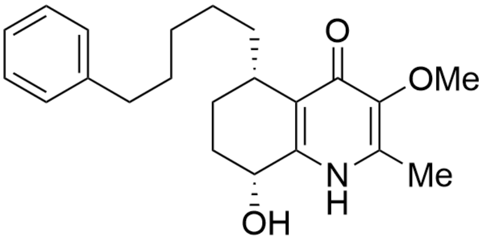	(8*R*)-8-hydroxy-waltherione M	*Waltheria indica*[84]

**101**	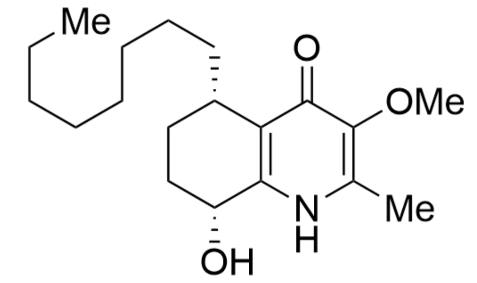	waltherione N	*Waltheria indica*[24]
**137**	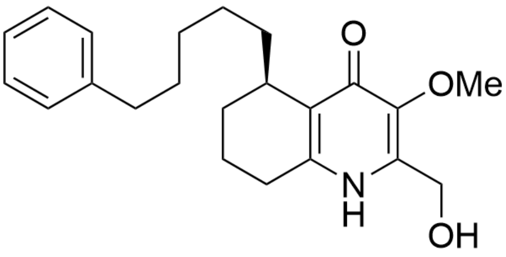	waltherione O	*Waltheria indica*[24]
**138**	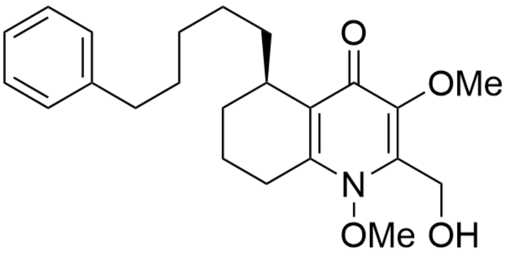	1-methoxy-waltherione O	*Waltheria indica*[84]
**119**	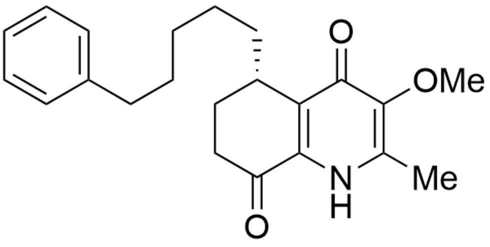	waltherione P	*Waltheria indica*[6,16,24]
**103**	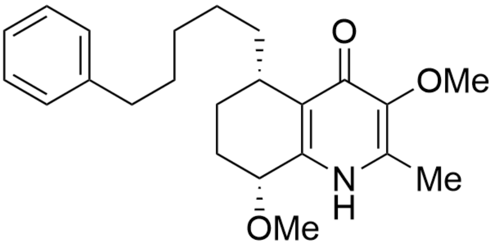	waltherione Q	*Waltheria indica*[10,24]
**139**	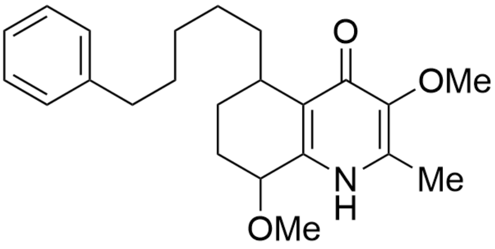	waltherione R	*Waltheria indica*[84]

**112**	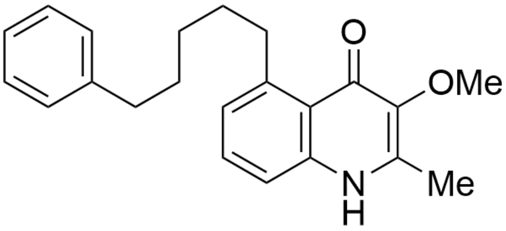	8-demethoxy-waltherione R	*Waltheria indica*[16]
**140**	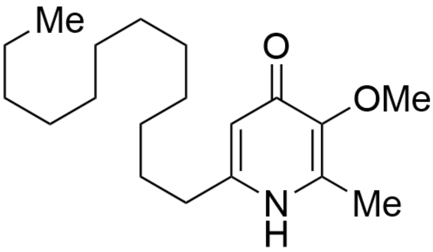	waltherione S	*Waltheria indica*[84]
**141**	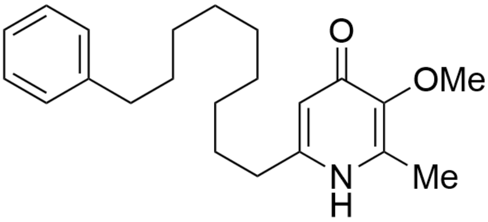	waltherione T	*Waltheria indica*[84]
**142**	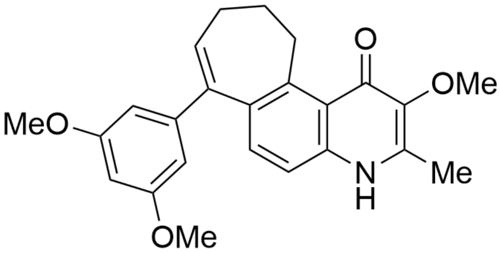	waltherione U	*Waltheria indica*[84]
**143**	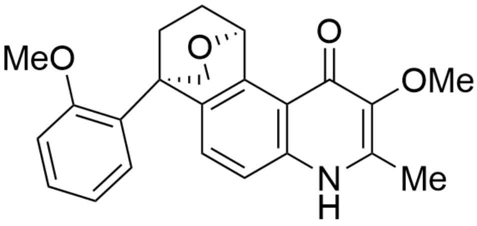	waltherione V	*Waltheria indica*[84]
**144**	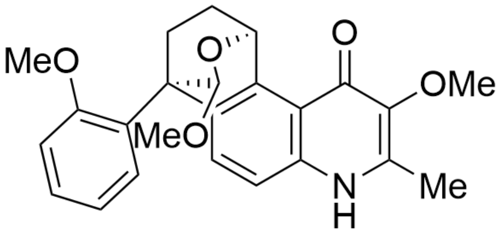	(*S*)-13-methoxy-waltherione V	*Waltheria indica*[84]

**7**	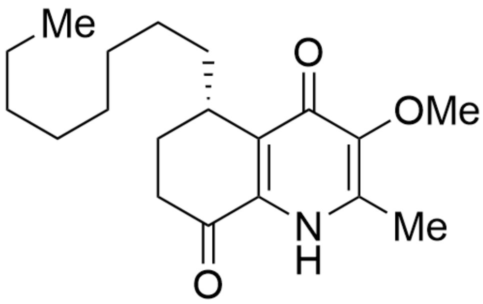	antidesmone	*Waltheria douradinha*[23] *Waltheria indica*[6,9,10,16,24,25]
**104**	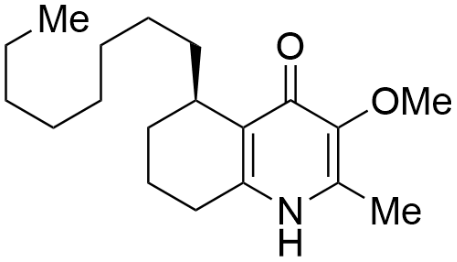	5(*R*)-8-deoxo-antidesmone	*Waltheria indica*[6,9,10,16,24]
**102**	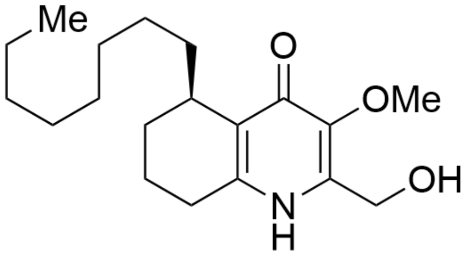	(*R*)-vanessine	*Waltheria douradinha*[23] *Waltheria indica*[24]
**10**	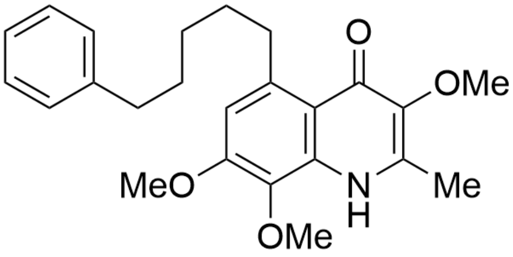	melovinone	*Waltheria indica*[84]
**11**	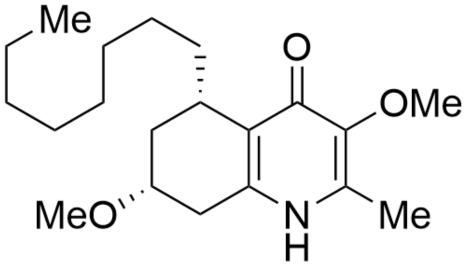	chamaedrone	*Waltheria indica*[6] *Waltheria brachypetala*[26]
**145**	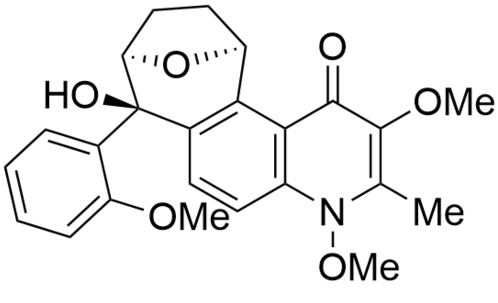	*N*-methoxy-waltherione A	*Waltheria indica*[6]

**146**	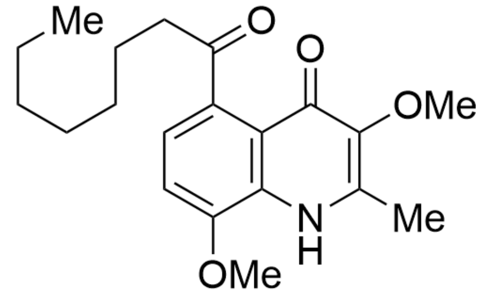	walindicaone A	*Waltheria indica*[16]
**147**	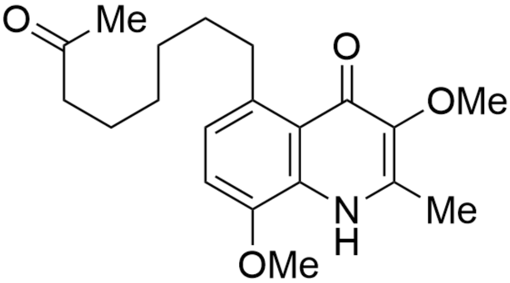	walindicaone B	*Waltheria indica*[16]
**148**	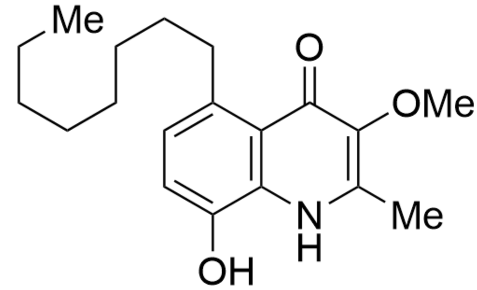	walindicaone C	*Waltheria indica*[16]
**149**	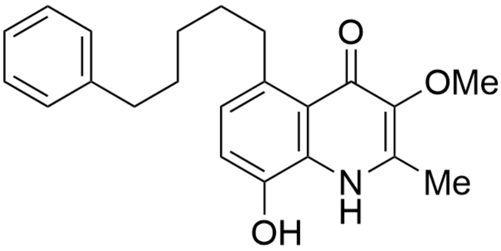	walindicaone D	*Waltheria indica*[16]
**150**	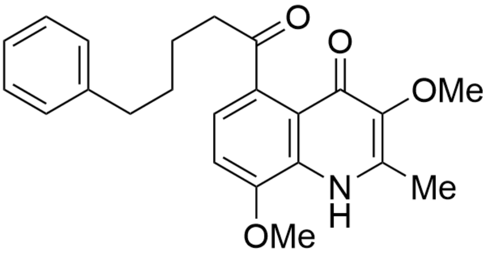	walindicaone E	*Waltheria indica*[16]
**114**	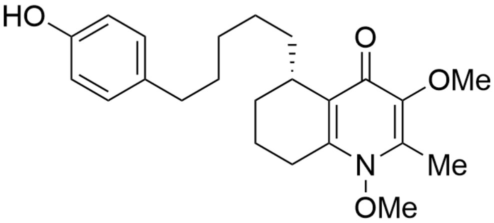	walindicaone F	*Waltheria indica*[16]

**115**	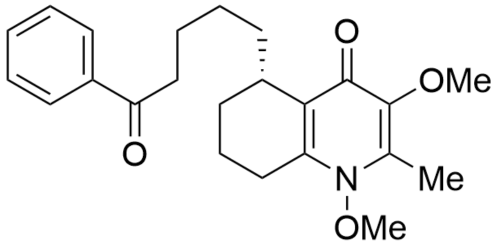	walindicaone G	*Waltheria indica*[16]
**116**	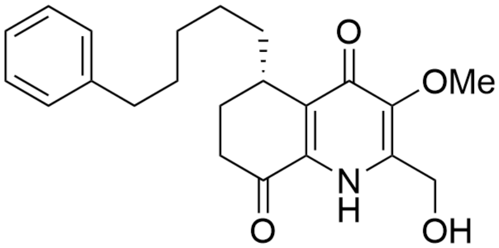	walindicaone H	*Waltheria indica*[16]
**151**	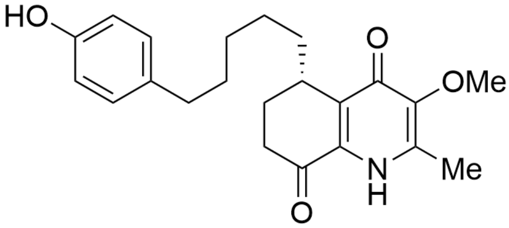	walindicaone I	*Waltheria indica*[16]
**152**	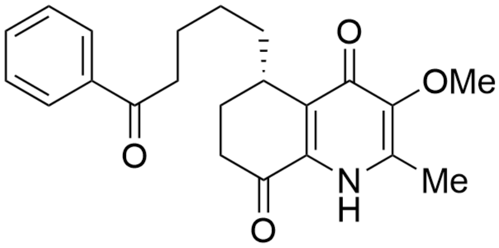	walindicaone J	*Waltheria indica*[16]
**153**	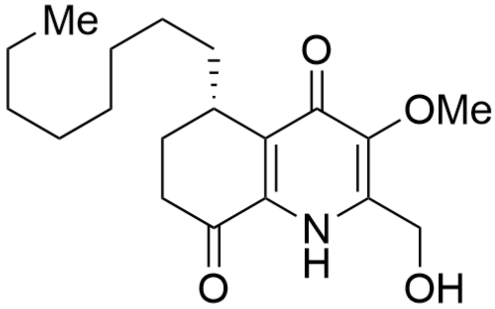	walindicaone K	*Waltheria indica*[16]
**154**	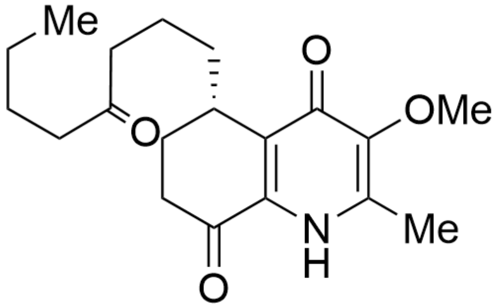	14-oxo-antidesmone	*Waltheria indica*[85]

**155**	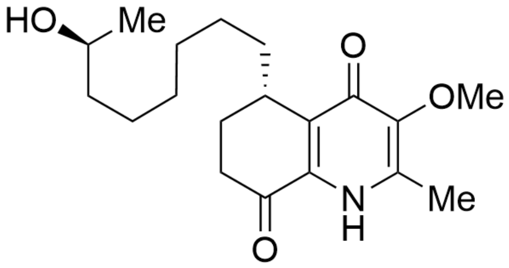	(*R*)-17-hydroxyantidesmone	*Waltheria indica*[85]
**156**	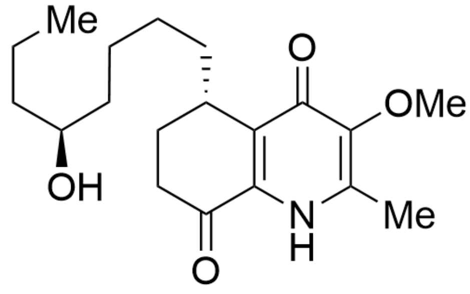	(*R*)-15-hydroxyantidesmone	*Waltheria indica*[85]
**157**	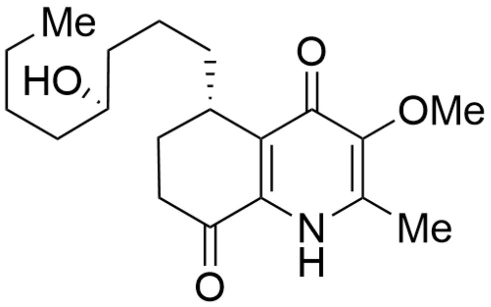	(*R*)-14-hydroxyantidesmone	*Waltheria indica*[85]
**158**	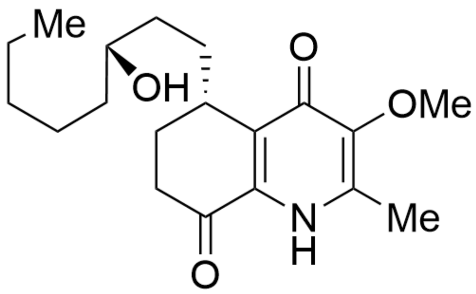	(*R*)-13-hydroxyantidesmone	*Waltheria indica*[85]
**159**	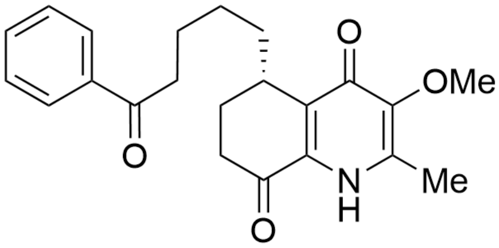	15-oxo-waltherione P	*Waltheria indica*[85]

**160**	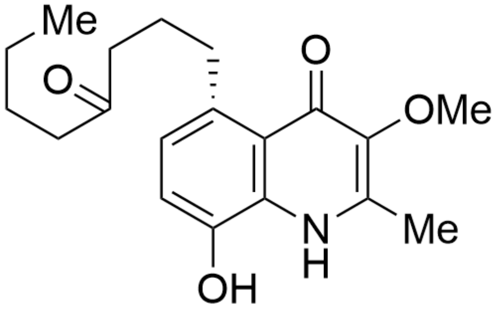	8-demethyl-14-oxo-waltherione F	*Waltheria indica*[85]
**161**	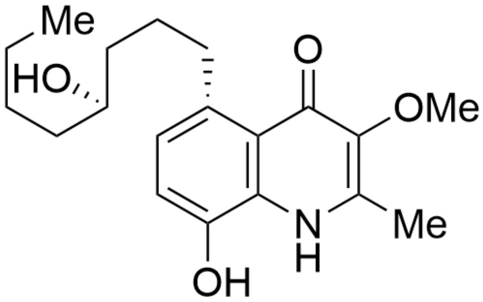	(*R*)-8-demethyl-14-hydroxywaltherione F	*Waltheria indica*[85]
**162**	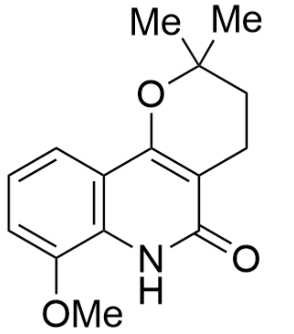	8-methoxyflindersine	*Waltheria brachypetala*[26]

Table S2. Cyclopeptide alkaloids from species of *Waltheria*.

**Compound**	**Structure**	**Name**	**Species**
**14**	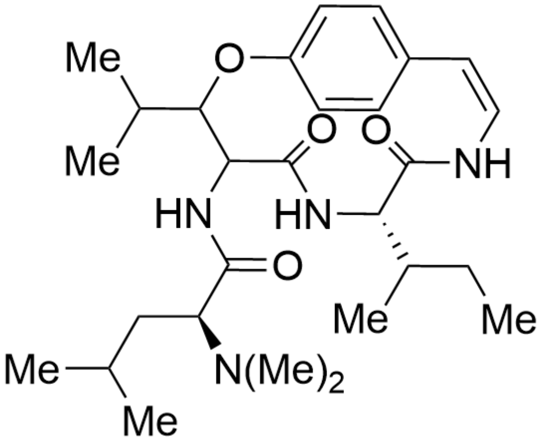	adouetine X	*Waltheria americana*[32,38]

**15**	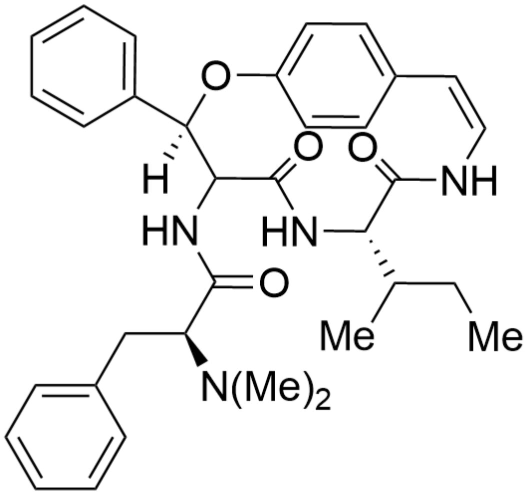	adouetine Y	*Waltheria americana*[32,38]
**163**	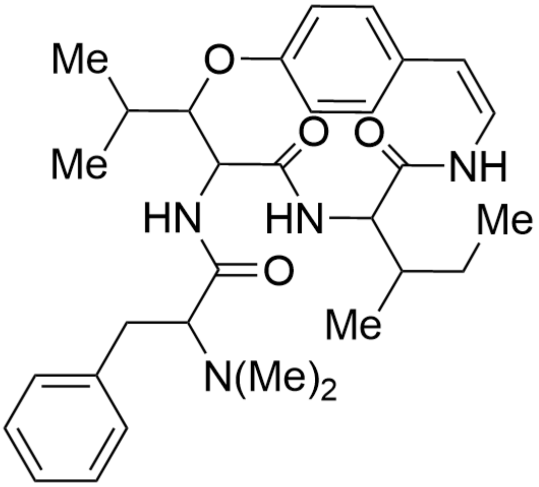	adouetine Y′	*Waltheria americana*[32,38] *Waltheria douradinha*[86]
**16**	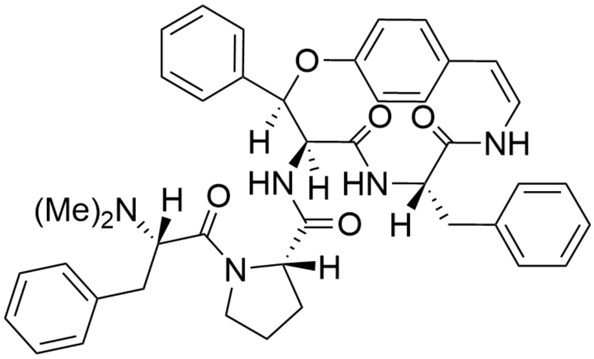	adouetine Z	*Waltheria americana*[32,38]
**111**	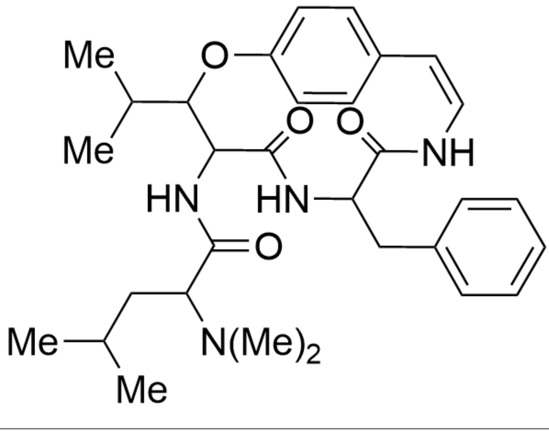	waltherine A	*Waltheria douradinha*[77,86] *Waltheria brachypetala*[28]

**164**	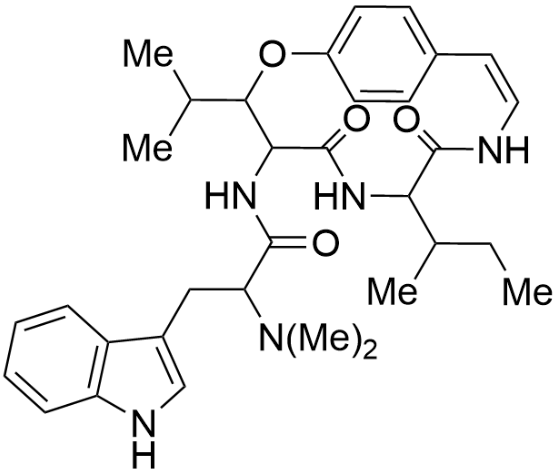	waltherine B	*Waltheria douradinha*[77,86]
**165**	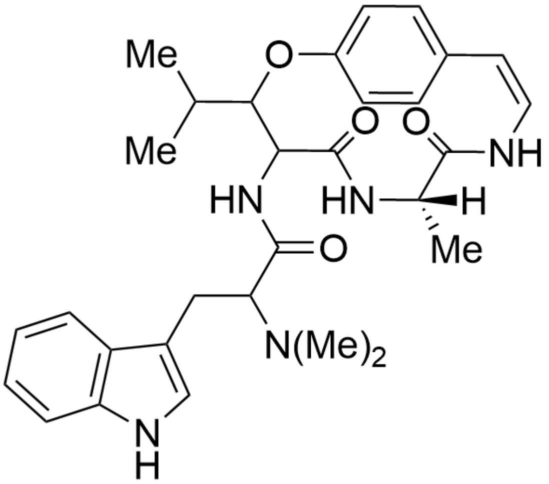	waltherine C	*Waltheria douradinha*[77]

Botanical information

The genus *Waltheria* L. has, among accepted names and synonyms, 80 species (See **List S1, Supplementary material**)[1–3]. Plants of this genus are characterized by being herbs, shrubs, subshrubs or small trees, of pantropical occurrence mainly in the hottest areas of the globe with 23 species native to Brazil. Plants of this genus have a prostrate to erect habit, with simple, petiolate leaves and jagged, serrated or crenate margins. Inflorescence terminal or axillary. Flowers small, reduced and usually yellowish [1,2,4].

Malvaceae family belongs to the Eudicotyledonous group, in the Rosideas clade and in Malvales order, according to the phylogenetic classification of Angiosperms. The large Malvaceae family corresponds to the union of four families: Malvaceae, Sterculiaceae, Bombacaceae and Tiliaceae [1,87,88]. The Malvaceae family Juss. is divided into nine subfamilies, namely: Bombacoideae, Brownlowioideae, Byttnerioideae, Dombeyoideae, Grewioideae, Helicteroideae, Malvoideae, Sterculioideae and Tilioideae. The genus *Waltheria* is inserted in the Byttnerioideae

subfamily, unlike the review presented by Viegas *et al*. (2022) which still classifies it in the old family to which it belonged, Sterculiaceae [4,5,87]. The Hermannieae tribe of the Malvaceae family is composed of four genera: *Hermannia*, *Dicarpidium*, *Melochia* and *Waltheria.* Of these, only *Melochia* and *Waltheria* have been studied in chemical terms, being reported as the main class of metabolites, the alkaloids [87]

List S1: Species of the genus *Waltheria*.

Waltheria acapulcensis Rose; *Waltheria ackermanniana* K. Schum.; *Waltheria acuminate* Rose (syn. *Waltheria alamosana* Standl.); *Waltheria albicans* Turcz.; *Waltheria arenaria* Ridl.; *Waltheria arenicola* A. Rodr.; *Waltheria astropus* Spreng. (syn. *Astropustomentosus* Spreng.); *Waltheria bahamensis* Britton; *Waltheria belizensis* J. G. Saunders; *Waltheria berteroi* (Spreng.) J. G. Saunders (syn. *Malachra berteroi* Spreng.; *Waltheria subcordata* Standl.); *Waltheria bicolor* J. G. Saunders; *Waltheria biribiriensis* [2]; *Waltheria brachypetala* Turcz.; *Waltheria bracteosa* A. St. Hil. & Naudin (syn. *Waltheria macropoda* Turcz.); *Waltheria calcicole* Urb.; *Waltheria capitata* Vell.; *Waltheria carmensarae* J. G. Saunders; *Waltheria carpinifolia* A. St. Hil. & Naudin; *Waltheria cinerascens* A. St. Hil.; *Waltheria cinerea* St. Hil. ex K. Schum.; *Waltheria collina* K. Schum.; *Waltheria communis* A. St. Hil. (syn. *Waltheria boliviensis* Turcz.; *Waltheria communis* var. *dietrichii* K. Schum.; *Waltheria communis* var. *erosa* Buxb.; *Waltheria communis* var. *gracilis* K. Schum.; *Waltheria communis* var. *henningsii* K. Schum.; *Waltheria communis* var. *hirta* K. Schum. & Hassl.; *Waltheria communis* var. *platyphylla* K. Schum.; *Waltheria communis* var. *tomentella* K. Schum.; *Waltheria communis* var. *velutina* K. Schum. & Hassl.; *Waltheria douradinha* A. St. Hil.; *Waltheria glabriuscula* A. St. Hil.; *Waltheria gracilis* A. St. Hil.; *Waltheria lanata* A. St. Hil.); *Waltheria conzattii* Standl.; *Waltheria cordata* Sm.; *Waltheria debilis* Bojer; *Waltheria detonsa* A. Gray; *Waltheria excels* Turcz.; *Waltheria fauriei* H. Lév.; *Waltheria ferruginea* A. St. Hil.; *Waltheria fruticose* Rottb.; *Waltheria fryxellii* J. G. Saunders; *Waltheria glabra* Poir. (syn. *Riedlea Berteroana* Balb. ex DC.; *Visenia berteroana* Spreng.; *Waltheria laevis* Wolf ex Schrank; *Waltheria ovalifolia* Urb.); *Waltheria glazioviana* K. Schum.; *Waltheria glomerata* C. Presl (syn. *Waltheria brevipes* Turcz.; *Waltheria rhombifolia* Donn. Sm.); *Waltheria incana* A. Chev.; *Waltheria incana* Ruiz & Pav. ex J. F. Macbr.; *Waltheria indica* L. (syn. *Waltheria africana* Schumach. & Thonn.; *Waltheria americana* L.; *Waltheria americana* var. *densiflora* K. Schum.; *Waltheria americana* var. *elliptica* K. Schum.; *Waltheria americana* var. *glandulosa* R. E. Fr.; *Waltheria americana* var. *indica* (L.) K. Schum.; *Waltheria americana* var. *subspicata* K. Schum.; *Waltheria angustifolia* L.; *Waltheria arborescens* Cav.; *Waltheria corchorifolia* Pers.; *Waltheria elliptica* Cav.; *Waltheria erioclada* DC.; *Waltheria guineensis* Schumach. & Thonn.; *Waltheria indica* var. *americana* (L.) R. Br. Ex Hosaka; *Waltheria indica* var. *prostrata* (K.Schum.) M. Gómez; *Waltheria laxa* Thulin; *Waltheria makinoi* Hayata; *Waltheria martii* Colla; *Waltheria prostrata* K. Schum.; *Waltheria wildii*

Suess.; *Waltheria involucrate* Benth. (syn. *Sitella involucrata* (Benth.) L. H. Bailey); *Waltheria ladewii* Rusby; *Waltheria lanceolata* R.Br. ex Mast.; *Waltheria lantanaefolia* St. Hilaire & Naudin; *Waltheria lantanifolia* A. St. Hil. & Naudin; *Waltheria longifolia* DC.; *Waltheria longiramea* Turcz.; *Waltheria lophantha* St. Lag.; *Waltheria lophanthus* G. Forst.; *Waltheria lundelliana* J. G. Saunders; *Waltheria macrophylla* Hassl.; *Waltheria madagascariensis* Hochr. (syn. *Visenia madagascarensis* Spreng.); *Waltheria marielleae* [3]; *Waltheria maritima* A. St. Hil.; *Waltheria melochioides* Loefl.; *Waltheria microphylla* Cav. (syn. *Melochia nipensis* Britton; *Waltheria intricate* Turcz.; *Waltheria nipensis* (Britton) Alain); *Waltheria mollis* Willd. ex K. Schum.; *Waltheria monogynia* Vell.; *Waltheria obtuse* Willd. ex Steud.; *Waltheria operculate* Rose (syn. *Turner avalleana* Standl. & L. O. Williams); *Waltheria ovate* Cav. (syn. *Waltheria ovata* f. *intermedia* (B. L. Rob.) Svenson; *Waltheria ovata* f. *reticulata* (Hook. f.) Svenson; *Waltheria reticulata* Hook. f.; *Waltheria reticulata* f. *acamata* B. L. Rob.; *Waltheria reticulata* f. *anderssonii* B. L. Rob.; *Waltheria reticulata* f. *intermedia* B. L. Rob.; *Waltheria sericea* Turcz.); *Waltheria paniculate* Benth.; *Waltheria pauciflora* Hochst. ex Mast.; *Waltheria pedunculata* Willd. ex Steud.; *Waltheria pentagynia*Vell.; *Waltheria petiolate* K. Schum.; *Waltheria pohliana* K. Schum.; *Waltheria polyantha* K. Schum.; *Waltheria preslii* Walp. (syn. *Waltheria haenkeana* D. Dietr.); *Waltheria pringlei* Rose & Standl.; *Waltheria procumbens* J. G. Saunders & Soria; *Waltheria pyrolifolia* A. Gray; *Waltheria regnellii* K. Schum. ex R. E. Fr.; *Waltheria rotundifolia* Schrank; *Waltheria scabra* (Colla) P. L. R. Moraes & Guglielmone (syn. *Viseniascabra* Colla; *Waltheria aspera* K. Schum.); *Waltheria selloana* K. Schum.; *Waltheria surinamensis* Turcz.; *Waltheria terminans*[2]; *Waltheria tomentosa* (J. R. Forst. & G. Forst.) H. St. John; *Waltheria tridentate* J. G. Saunders; *Waltheria vernonioides* R. E. Fr.; *Waltheria virgate* Ewart & Cookson and *Waltheria viscosissima* A. St. Hil. (syn. *Waltheria hirsuta* C. Presl; *Waltheria machrisiana* L. B. Sm.; *Waltheria tubiflora* Klotzsch).

Traditional names for *Waltheria indica*

Waltheria indica, also known as velvet leaf, monkey bush, marshmallow, boater bush, buff coat, and leather coat, has a wide range of local names reflecting its global distribution. The species name "*indica*" associates it with India, where it is called 'Nallabenda' in Telugu and 'Shengali-poondu' in Tamil. In northern Nigeria, it is known as 'kafafi' in Fulani, 'hankufah' or 'hankubah' in Hausa, 'efu-abe' in Nupe, and 'korikodi' in Yoruba. In other regions, it is referred to as 'matum kevel' in Wolof, 'kiaza' or 'kerza' in Bissa, 'yar-yamde' in Moore, and 'Mokhutesela' in South Africa's Limpopo province. In Mexico, it is called 'güinar' and 'manrubio o tapacola', in Panama 'hierba de soldado', in the Philippines 'barulad', and in Hawaii 'hi'aloa', 'uhaloa', 'kanakaloa', 'mauve-gris', 'motobranco', 'fulutafu', and 'kafaki'. Spanish names include 'basora-prieta', 'escobilloblanco', and 'malvavisco' [89]. In Brazil it is known as 'vassourinha' (small broom) [6].
